# Identification of VEGFs-related gene signature for predicting microangiogenesis and hepatocellular carcinoma prognosis

**DOI:** 10.18632/aging.205931

**Published:** 2024-06-13

**Authors:** Shengpan Jiang, Guoting Zhu, Yiqing Tan, Tao Zhou, Shilin Zheng, Fuhua Wang, Wenfeng Lei, Xuan Liu, Jinjun Du, Manman Tian

**Affiliations:** 1 Department of Interventional Medicine, Wuhan Third Hospital (Tongren Hospital of Wuhan University), Wuhan, Hubei Province, China; 2 Department of Neurosurgery, Renmin Hospital of Wuhan University, Wuhan, Hubei Province, China; 3 Department of Hepatology and Gastroenterology, Wuhan Hospital of Traditional Chinese Medicine (The Third Clinical College of Hubei University of Chinese Medicine), Wuhan, Hubei Province, China

**Keywords:** hepatocellular carcinoma, microangiogenesis, VEGF, immune cell, gene signature

## Abstract

Microangiogenesis is an important prognostic factor in various cancers, including hepatocellular carcinoma (HCC). The Vascular Endothelial Growth Factor (VEGF) has been shown to contribute to tumor angiogenesis. Recently, several studies have investigated the regulation of VEGF production by a single gene, with few researchers exploring all genes that affect VEGF production. In this study, we comprehensively analyzed all genes affecting VEGF production in HCC and developed a risk model and gene-based risk score based on VEGF production. Moreover, the model’s predictive capacity on prognosis of HCCs was verified using training and validation datasets. The developed model showed good prediction of the overall survival rate. Patients with a higher risk score experienced poor outcomes compared to those with a lower risk score. Furthermore, we identified the immunological causes of the poor prognosis of patients with high-risk scores comparing with those with low-risk scores.

## INTRODUCTION

Primary hepatocellular carcinoma (HCC) is a highly invasive malignant tumor posing a significant burden on healthcare globally. Its 5-year survival rate is less than 21% [1]. HCC is the main type of liver cancer, accounting for 90% of all reported liver cancer cases and the fourth leading cause of cancer deaths worldwide [2]. The development of HCC is driven by several factors, among which angiogenesis is the most important biological feature considered to be a key treatment target [3]. Angiogenesis is a process in which new blood vessels are formed from existing ones to build new vascular networks. Angiogenesis have been implicated in numerous physiological processes, like the tissue repair, embryonic development and inflammatory response. However, in pathological situations, such as tumors, excessive angiogenesis provides nutrients and oxygen to tumor cells thereby promoting tumor growth, invasion, and metastasis [4–7]. HCC is a highly angiogenic tumor, and the level of its angiogenesis correlates with the degree of the tumor stage, grade, prognosis, and therapeutic efficacy [8].

Angiogenesis is a complex process involving multiple cellular and molecular interactions. In this process, vascular endothelial growth factor (VEGF) functions as a crucial role, which can bind to its receptor (VEGFR), thereby activating multiple signaling pathways to regulate the functions of vascular endothelial cells, such as proliferation, migration, lumen formation, and vascular permeability, etc. [9–12]. VEGF expression in HCC is modulated by various factors, such as hypoxia, inflammation, tumor microenvironment, and gene mutations [13–15]. VEGF is the dominant factor influencing HCC angiogenesis, and its level is closely associated with the degree of malignancy, metastatic ability and prognosis of HCC [3, 16, 17]. In addition, VEGF not only regulates HCC angiogenesis, but also affects the biological characteristics of HCC cells, like the proliferation, apoptosis, invasion and metastasis [18, 19]. Therefore, VEGF is an important molecular marker and therapeutic target for HCC, and drugs targeting VEGF or VEGFR have shown promising potential in the clinical treatment of HCC [20–23].

To date, several prognostic prediction models based on angiogenesis-related genes have been developed for HCC, but no study has investigated this key factor of HCC angiogenesis in detail. Moreover, the role of VEGF in hepatocellular carcinoma angiogenesis is not fully understood [24–27]. Moreover, considering the heterogeneity and complexity of hepatocellular carcinoma, the detection and inhibition of VEGF or VEGFR alone may not be sufficient to reflect and predict the angiogenic status and therapeutic efficacy of HCC [28]. Therefore, it is necessary to explore the gene expression profiles of VEGF from a global perspective to build a gene signature that might comprehensively assess and predict microangiogenesis and prognosis of HCC patients. For our design, we constructed a gene signature capable of predicting microangiogenesis and prognosis of HCC by screening genes associated with VEGF production. Its clinical significance and biological function in HCC were validated.

## MATERIALS AND METHODS

### Public data and collection of samples

Clinical, molecular, and whole-genome RNA-seq expression data were downloaded from TCGA database (https://portal.gdc.cancer.gov/). After exclusion of samples without prognostic information or incomplete clinical information, 374 HCC samples were included. In addition, the TCGA database was used to obtain whole-genome RNA-seq expression data of 50 normal liver samples. A total of 232 HCC patient samples were retrieved from International Cancer Genome Consortium (ICGC) database (https://dcc.icgc.org/). Moreover, the GSE76427, which includes the expression and corresponding clinical data of another 114 HCC patient samples, was gained from the Gene Expression Omnibus (GEO) database (https://www.ncbi.nlm.nih.gov/geo/). The samples were merged and standardized to create validation sets. We excluded cases with less than or exactly 10 days of survival or patients without survival data since they could have died from unrelated complications. In such cases, 50 normal liver samples and 310 HCC samples from TCGA, 231 HCC samples from ICGC, and 114 HCC samples from GSE76427, were selected for subsequent analyses. Furthermore, we considered the possibility of batch effects present among different databases as well as within the same database. To address this issue, “normalize between arrays” function [29, 30] of R package “limma” was utilized to remove multiple batch influences when merging the mRNA_seq data of ICGC, TCGA and GSE76427.

### Patient samples

The tissues were obtained with informed consent from all participating patients. Six control samples were collected between September 2019 and June 2022, five samples from patients with HCC and five samples from non-tumor liver tissues. Preoperative chemotherapy or radiotherapy was not used to treat any of the HCCs. Independent samples from our hospital were verified by the hub genes of GSRS mRNA levels in HCC.

### Obtaining vascular endothelial growth factor production-related gene sets

Gene sets, “GOBP_VASCULAR_ENDOTHELIAL_GROWTH_FACTOR_PRODUCTION”, were obtained from the Molecular Signatures Database (http://www.gsea-msigdb.org/gsea/index.jsp).

### Identifying differentially expressed genes between normal liver tissues and hepatocellular carcinoma

The training set was established using HCC and correlated normal liver samples from TCGA databases. DEGs were identified from Vascular endothelial growth factor Production-RGlated genes (VPRGs) using the R package “limma” [30]. The criterion set for this study was a *p*-value less than 0.05, with an abs value of logFC greater than 0.585.

### Protein-protein interaction network analysis

The STRING database (https://cn.string-db.org/) was used to construct the PPI network containing 34 VPRGs. Nodes with interaction confidences greater than 0.4 were displayed.

### Genomic alterations of 34 vascular endothelial growth factor production-related genes

The cBioPortal dataset (http://www.cbioportal.org/) was utilized to explore the CNV amplification, truncating mutation, in-frame mutation, missense mutation, CNV deep deletion, and fusions involving the 34 genes.

### Construction of vascular endothelial growth factor production-related risk signature

To assess the prognostic value of VPRGs in HCC, we conducted univariate Cox regression analysis with the “survival” R package. Genes with a p-value < 0.05 were selected for analysis. Related risk signature was then developed for HCC by incorporating survival status, survival time, and expression levels of prognosis-related genes. The Least Absolute Shrinkage and Selection Operator (LASSO) regression algorithm [31] was utilized, with the penalty parameter λ determined through 10-fold cross-validation. By identifying the genes and their respective regression coefficients, we determined the most appropriate λ value. A formula applied for this purpose was as follows: Riskscore = exprgene (1) × coefficient gene (1) + coefficient gene (2) × exprgene (2) + . . . + coefficient gene (n) ×exprgene (n)

here, n represents the prognostic genes, exprgene represents the coefficient gene. The coefficient of the gene in the risk signature represents the expression value of the gene.

### Principal components analysis

The samples were divided into low and high-risk groups based on the median risk scores. The Principal Component Analysis (PCA) was employed to clarify the variances among these two groups. The variation in mRNA expression data among the high and low-risk groups in TCGA-, ICGC- and GSE76427-HCC datasets were observed using dimensionality reduction.

### Prognostic analysis of vascular endothelial growth factor production-related risk signature

Both Kaplan–Meier survival curves and Cox regression analysis were applied to evaluate the prognostic significance of vascular endothelial growth factor production-related risk signature (VPRS) in HCC in training and verification datasets. The area under the ROC curves (AUC) was calculated for the predictive value of VPRS for the 1-, 3-, and 5-year Overall Survival (OS) in HCC patients. The diagnostic procedure’s precision was evaluated with the AUC-ROC (Area Under the Receiver Operating Characteristic curve), employing three defined categories based on [32]: low accuracy (0.5 < AUC-ROC ≤ 0.7), moderate accuracy (0.7 < AUC-ROC ≤ 0.9), and high accuracy (0.9 < AUC-ROC ≤ 1).

### Clinicopathological importance of the vascular endothelial growth factor production-related risk signature

Patients in both training and verification sets were classified as low and high-risk sets. The chi-square test was performed to investigate the correlation among the clinicopathological features and risk score, among them, age, gender, N classification, T classification, M classification, Eastern Cancer Oncology Group (ECOG), vascular invasion, tumor grade, and stage.

### Tumor-infiltrating immune cells profiles

The population of immune cells in low-risk and high-risk groups was estimated using the CIBERSORT computational method. In both the TCGA and ICGC-HCC cohorts, we utilized Pearson correlation analysis and the Wilcoxon test to examine the association between the fraction of Tumor-Infiltrating Immune Cells (TIICs) and the risk score. We then utilized the ESTIMATE algorithm, specifically the “estimate” package, to calculate the immune score, stromal score and tumor purity for each HCC sample [33].

### Single-sample gene sets enrichment analysis

All significant genes of the 29 immune-related pathways were obtained from a previous study [34]. The TIICs level was estimated by the single-sample GSEA (ssGSEA; [35]) using melanoma mRNA Tumor Mutation Burden (TMB) data. Moreover, the enrichment level of gene hallmarks for 29 hallmarks related to immune was analyzed in the training and verification cohorts, among the low and high-risk groups, with a significance threshold of *p*-value < 0.05. Additionally, given the significance of Immune Checkpoints (ICPs) and Immunogenic Cell Death (ICD) modulators in cancer immunity, the expression levels of these proteins were compared among the high and low-risk groups.

### Analysis of tumor mutational load

In the TCGA-HCC dataset, total tumor somatic mutations detected in each sample was used for computation. The prognostic value of TMB in HCC was also analyzed. The R package “maftools” was applied to calculate and compare the mutational status in high and low-risk groups.

### Analysis of hallmark gene sets

Further analysis focused on hallmark gene sets derived from the Molecular Signatures Database. These sets represent and summarize explicit, well-defined biological states or displays and processes that are coherently expressed. The R package “GSVA” was applied on low and high-risk groups in TCGA-HCC to implement GSVA of hallmark gene sets [36].

### Verification of hub genes of vascular endothelial growth factor production-related risk signature

In both training and validation datasets, the genes correlated with the prognosis of VPRS were evaluated with the Kaplan-Meier survival curves. For further investigation, the Human Protein Atlas (HPA) database (https://www.proteinatlas.org/) was adopted to assess the gene protein level identified in both normal liver and HCC tissues.

### Quantitative real-time PCR (qRT-PCR)

RNA was extracted from liver tissues using the TRIzol reagent (as described in [37]) for qRT-PCR analysis. SYBR Green assays and specific primers were used to quantify gene expression. Relative Ct values, normalized to GAPDH, were employed to compare gene expression between the experimental and control groups.

### Data availability statement

The authors will unreservedly share raw data that support the conclusions of this article.

## RESULTS

### Genetic alterations of vascular endothelial growth factor production-related genes in hepatocellular carcinoma

**Figure 1:**
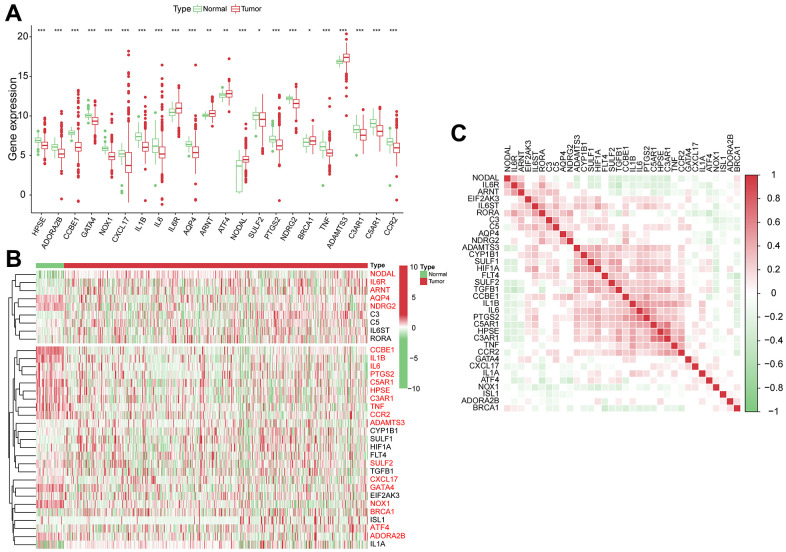
**The genomic characterization of VPRGs.** (**A**) Boxplot for differentially expressed VPRGs. (**B**) Heatmap for differentially expressed VPRGs; genes with red color are significantly differently expressed between normal liver tissues and HCC tissues. (**C**) Correlation plot for VPRGs; red and green squares indicate positive and inverse correlation respectively. ***p < 0.001, **p < 0.001, *p < 0.05.

We performed a differential analysis of 34 VPRGs in the training group and identified 22 DEGs between normal and HCC cases (Figure 1A, 1B). Supplementary Table 1 shows the upregulated and downregulated VPRGs with their analogous logFC values. In addition, Spearman’s correlation analysis was performed to characterize the expression correlations among the VPRGs and the results were presented in Figure 1C. A PPI network analysis also revealed a substantial co-expression correlation among the VPRGs, as demonstrated in Supplementary Figure 1A. Additionally, for further understanding of the genomic characteristics of VPRGs in HCC, we analyzed somatic mutational status and the copy number variation of GSRGs using the cBioPortal database (Supplementary Figure 1B).

### Construction and verification of vascular endothelial growth factor production-related risk signature

**Figure 2:**
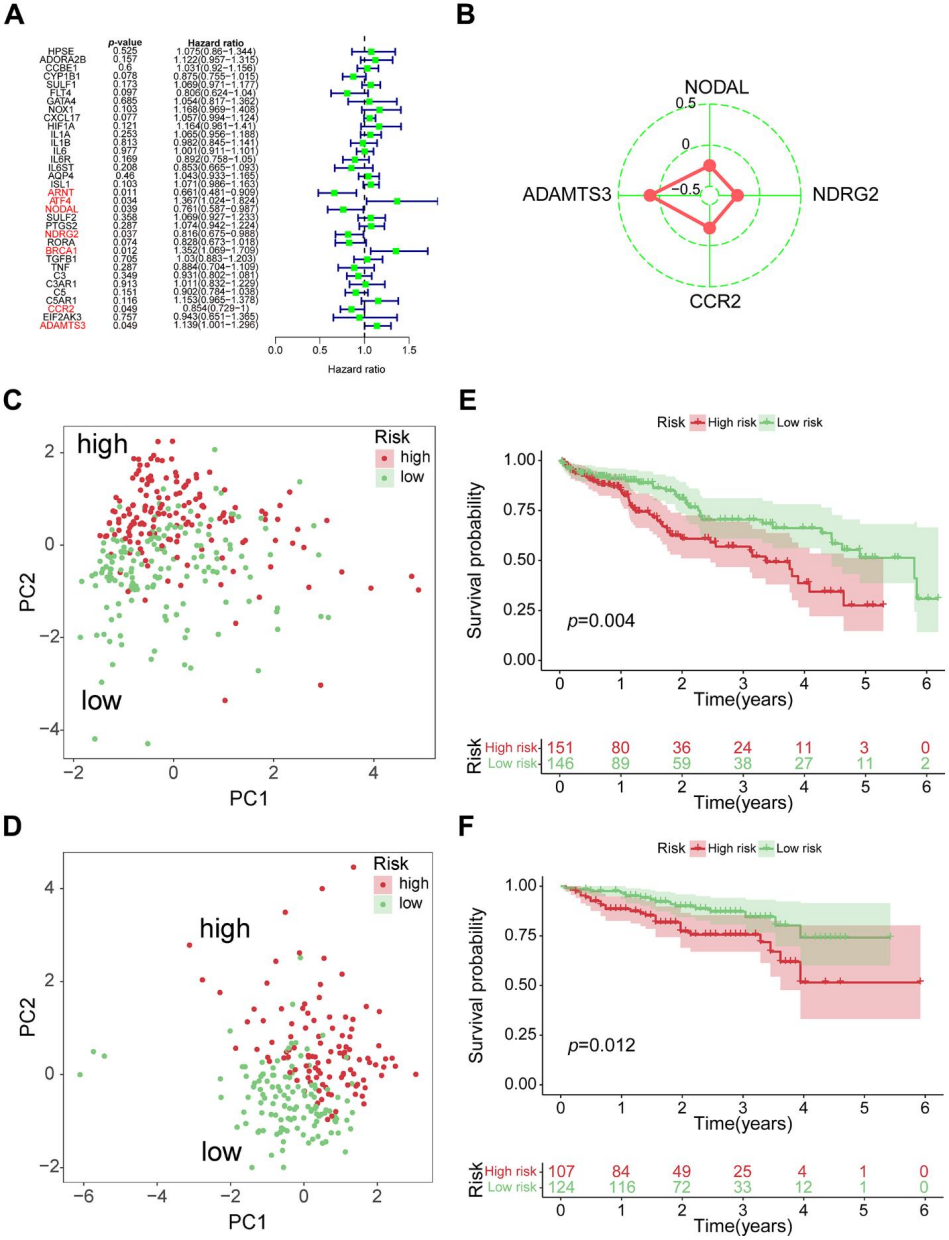
**Construction of 4-genes VPRS.** (**A**) Forest plot for the survival analysis of HCC patients with a univariate Cox model after adjustment for VPRGs; red color represents *p* < 0.05. (**B**) Radar diagram of efficiency of the 4 genes in VPRS; the closer the red dot is to the outside, the greater the value it represents. (**C**) PCA of HCC samples in TCGA; dots in red and green represent samples in high-risk and low-risk groups respectively. (**D**) Overall survival analysis of risk score for HCC patients in TCGA. (**E**) PCA in ICGC-HCC. (**F**) Survival analysis in ICGC-HCC.

From 34 VPRGs, univariate Cox analysis identified 7 genes significantly correlated with prognosis (p < 0.05) (Figure 2A). LASSO regression further narrowed this down to 4 key genes: *ADAMTS3, CCR2, NDRG2,* and *NODAL* (Supplementary Figure 2A, 2B). A risk score was then calculated for each patient based on the expression levels and coefficients of these 4 genes (Figure 2B). The PCA results further revealed significant separation between high- and low-risk groups based on the median VPRS in both training and validation sets (Figure 2C, 2D and Supplementary Figure 2C).

Survival analysis demonstrated significantly different clinical outcomes between the high- and low-risk groups in both training and validation sets (Figure 2E, 2F and Supplementary Figure 2D). Notably, the VPRS across TCGA, ICGC, and GSE76427-HCC datasets exhibited distinct patterns in risk gene expression, risk scores, and survival states, highlighting the VPRS’s potential as a valuable prognostic predictor compared to other clinical factors (Supplementary Figure 2E–2G). Supplementary Table 2 summarizes the 4 genes and their corresponding coefficients in the optimal model. Altogether, above results demonstrated that the risk score based on VPRS could be a better indicator for predicting the prognosis of HCC compared with other clinical factors.

### The risk score acts as an independent factor for predicting overall survival of hepatocellular carcinoma patients

**Figure 3:**
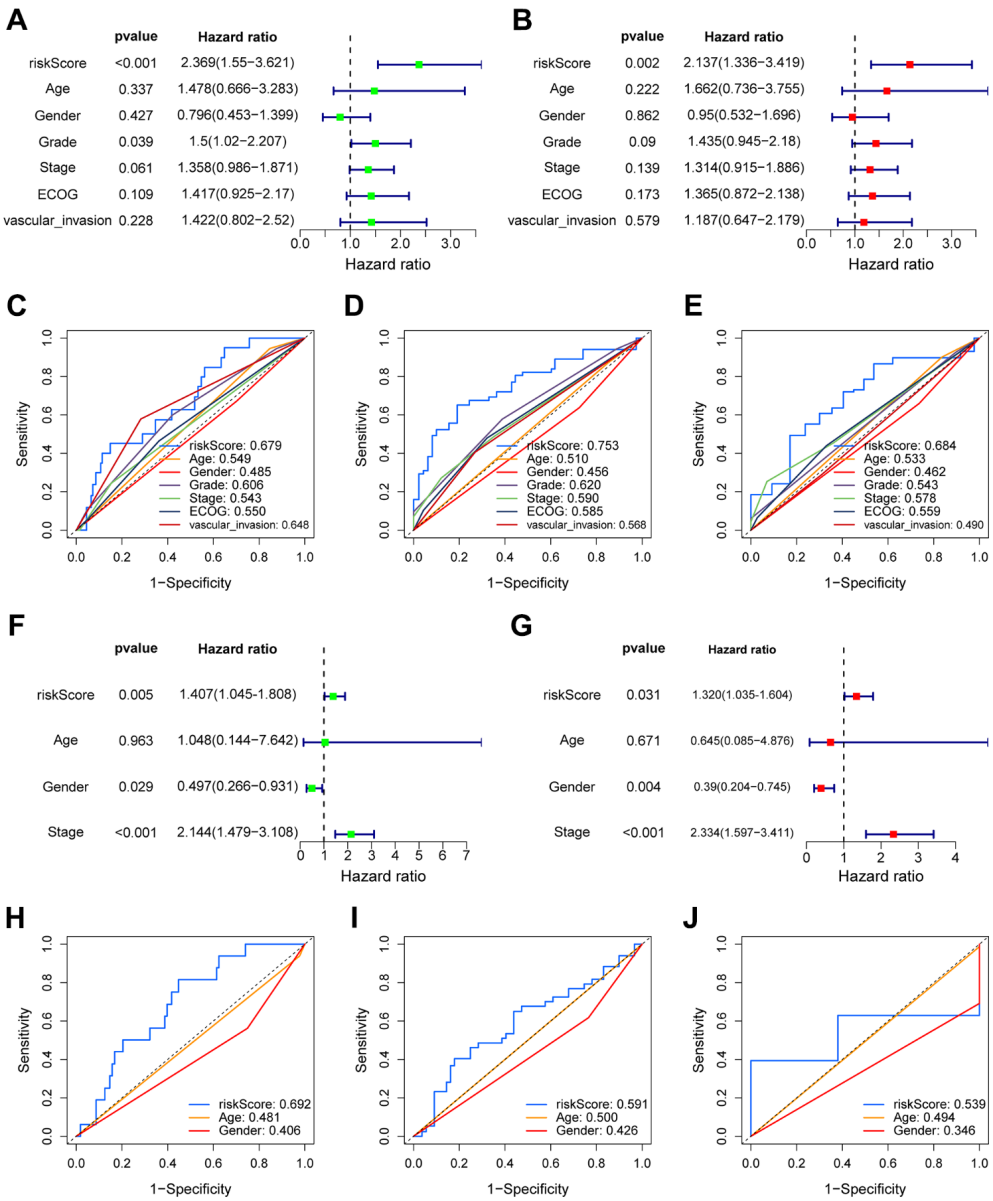
**The prognostic value of VPRS.** In the training set, forest plot on the left for the univariate Cox test (**A**) evaluating the correlation of the risk score and clinical factors with OS of patients, and forest plot on the right for the multivariate Cox analysis (**B**) identifying independent risk factors for the OS of patients. The ROC curve of risk score and clinical factors to predict 1- (**C**), 3- (**D**), and 5-year (**E**) OS. In the validation set, univariate (**F**) and multivariate (**G**) COX analysis of risk score and clinical factors. ROC curve of risk score compared with other clinical factors to predict 1- (**H**), 3- (**I**), and 5-year (**J**) OS.

We conducted both univariate and multivariate COX analysis to assess the independent prognostic value of the VPRS to predict the outcomes of HCC patients. In the TCGA-HCC group, the risk score independently predicted the OS (overall survival) (HR = 2.137, *p* = 0.002) (Figure 3A, 3B). Additionally, the risk score exhibited a higher AUC-ROC unlike additional clinical features, including age, gender, ECOG, vascular invasion, tumor grade, and stage. The AUC of the risk score in the training group was 0.679, 0.753, and 0.684 for 1-, 3-, and 5-year OS of patients, respectively (Figure 3C–3E). The findings were also validated in the ICGC- and GSE76427-HCC cohort. In multivariate COX regression, the risk score had an HR of 1.320 in ICGC (Figure 3F, 3G, *p* = 0.031), while it was 1.226 in GSE76427 (Supplementary Figure 3A, 3B, *p* = 0.039). Moreover, the AUC values of 1-, 3-, and 5-year OS of patients in ICGC-HCC were 0.692, 0.591, and 0.539, respectively (Figure 3H–3J), while there were 0.474, 0.419, and 0.473 in GSE76427-HCC, respectively (Supplementary Figure 3C–3E).

### Relationship between vascular endothelial growth factor production-related risk signature and the features of clinicopathology

**Figure 4:**
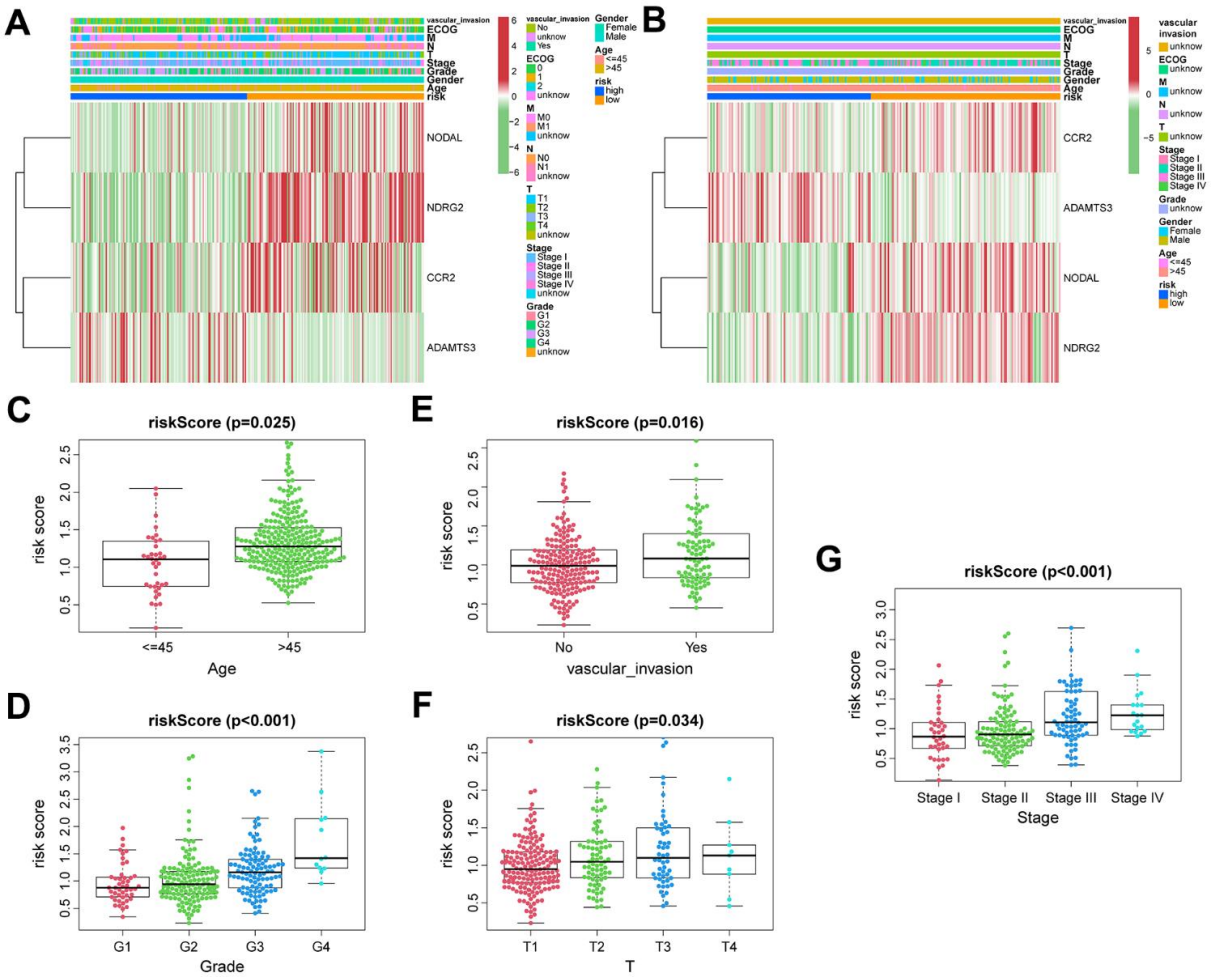
**The correlation between clinicopathological factors and risk score.** Heatmap of the correlations between clinicopathological characteristics of HCC and risk score in the TCGA (**A**) and ICGC (**B**) cohorts. Distribution of vascular endothelial growth factor production-related risk signature within HCC patients stratified by age, tumor grade, T classification, and vascular invasion in TCGA (**C**–**F**) and tumor stage in ICGC (**G**) cohorts.

During the training phase, we identified a total of 310 cases that included clinical data such as age, gender, T classification, N classification, M classification, ECOG, vascular invasion, tumor grade, and stage (Figure 4A). In the ICGC sets, we narrowed down the cases to 231 by considering only those with clinical features of age, gender, and tumor stage (Figure 4B). To compare the distribution of clinical characteristics among different risk groups, we utilized the “chisq.test” function in R to conduct Chi-square tests. The results of these tests for both the TCGA and ICGC cohorts can be found in Table 1. Based on VPRS, the risk scores substantially correlated with age, tumor grade, and T classification in TCGA cohorts (Figure 4C–4F). Furthermore, we noted a major correlation between the risk scores and tumor stage in ICGC groups (Figure 4G). HCC samples with higher age, T classification, tumor grade, and stage specifically exhibited markedly higher risk scores than those with lower values, with risk score and vascular invasion also showing a significant correlation. Thus, the risk score values were significantly correlated with age, T classification, vascular invasion, tumor grade, and stage of HCC.

**Table 1:** Correlation between clinicopathological factors of hepatocellular carcinoma patients and 4-VPRS genes risk scores in the two cohorts.

**Features**	**Training set TCGA RNA-seq cohort (n = 310)**			**Validation set ICGC RNA-seq cohort (n = 231)**		
	**Low-risk score** **(n = 155)**	**High-risk score** **(n = 155)**	***P*-value**	**Low-risk score** **(n = 124)**	**High-risk score** **(n = 107)**	***P*-value**
**Age**			0.025			0.778
≤ 45	14	24		3	3	
> 45	141	131		121	104	
**Gender**			0.963			0.375
Female	51	48		35	26	
Male	104	107		89	81	
**Grade**			<0.001			-
G1	30	16		-	-	
G2	82	64		-	-	
G3	32	69		-	-	
G4	7	5		-	-	
unknow	4	1		-	-	
**Stage**			0.06			<0.001
Stage I	87	75		19	17	
Stage II	34	37		70	36	
Stage III	21	29		26	44	
Stage IV	1	2		9	10	
unknow	12	12		0	0	
**T**			0.034			-
T1	92	79		-	-	
T2	34	41		-	-	
T3	22	30		-	-	
T4	4	5		-	-	
unknow	3	0		-	-	
**N**			0.109			-
N0	97	109		-	-	
N1	0	3		-	-	
unknow	58	43		-	-	
**M**			0.918			-
M0	99	114		-	-	
M1	1	2		-	-	
unknow	55	39		-	-	
**ECOG**			0.623			-
0	77	82		-	-	
1	42	40		-	-	
2	8	6		-	-	
unknow	28	27		-	-	
**vascular invasion**			0.016			-
Yes	39	50		-	-	
No	97	95		-	-	
unknow	19	10		-	-	

### Tumor-infiltrating immune cells profiles

**Figure 5:**
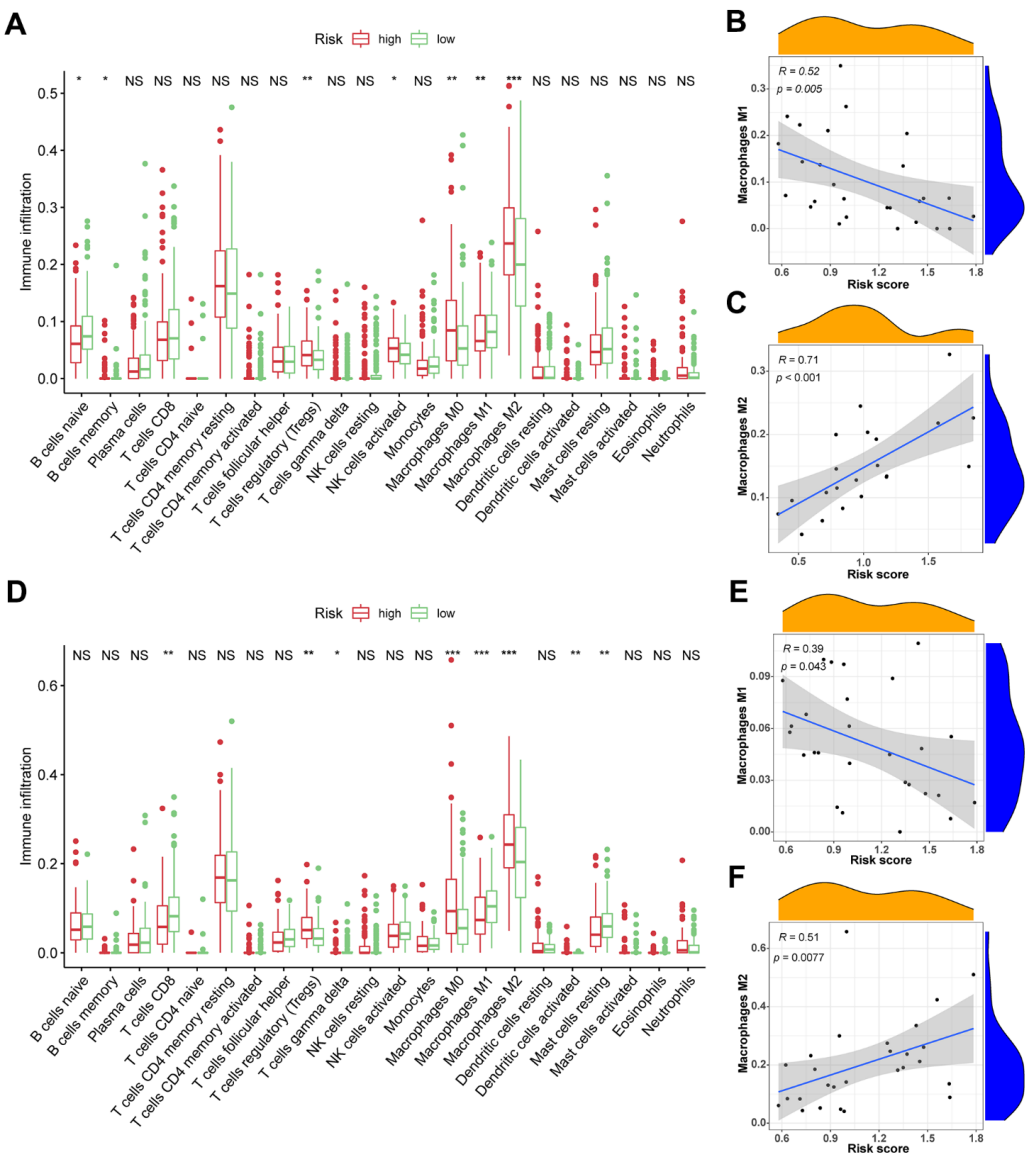
**The association between TIICs and VPRS.** (**A**) Differential analysis of 22 kinds of TIICs in the low and high-risk groups in training sets. (**B**, **C**) Spearman’s correlation analysis for risk score and M1 and M2 macrophages in TCGA cohorts, each dot plot represents a subject, and the correlation is fitted into a straight blue line. (**D**) Differential analysis of 22 kinds of TIICs in the low and high-risk groups invalidation sets. (**E**, **F**) Spearman’s correlation analysis for risk score and M1 and M2 macrophages in ICGC cohorts, each dot plot represents a subject, and the correlation is fitted into a straight blue line. R, rho; ***p < 0.001, **p < 0.001, *p < 0.05.

**Figure 6:**
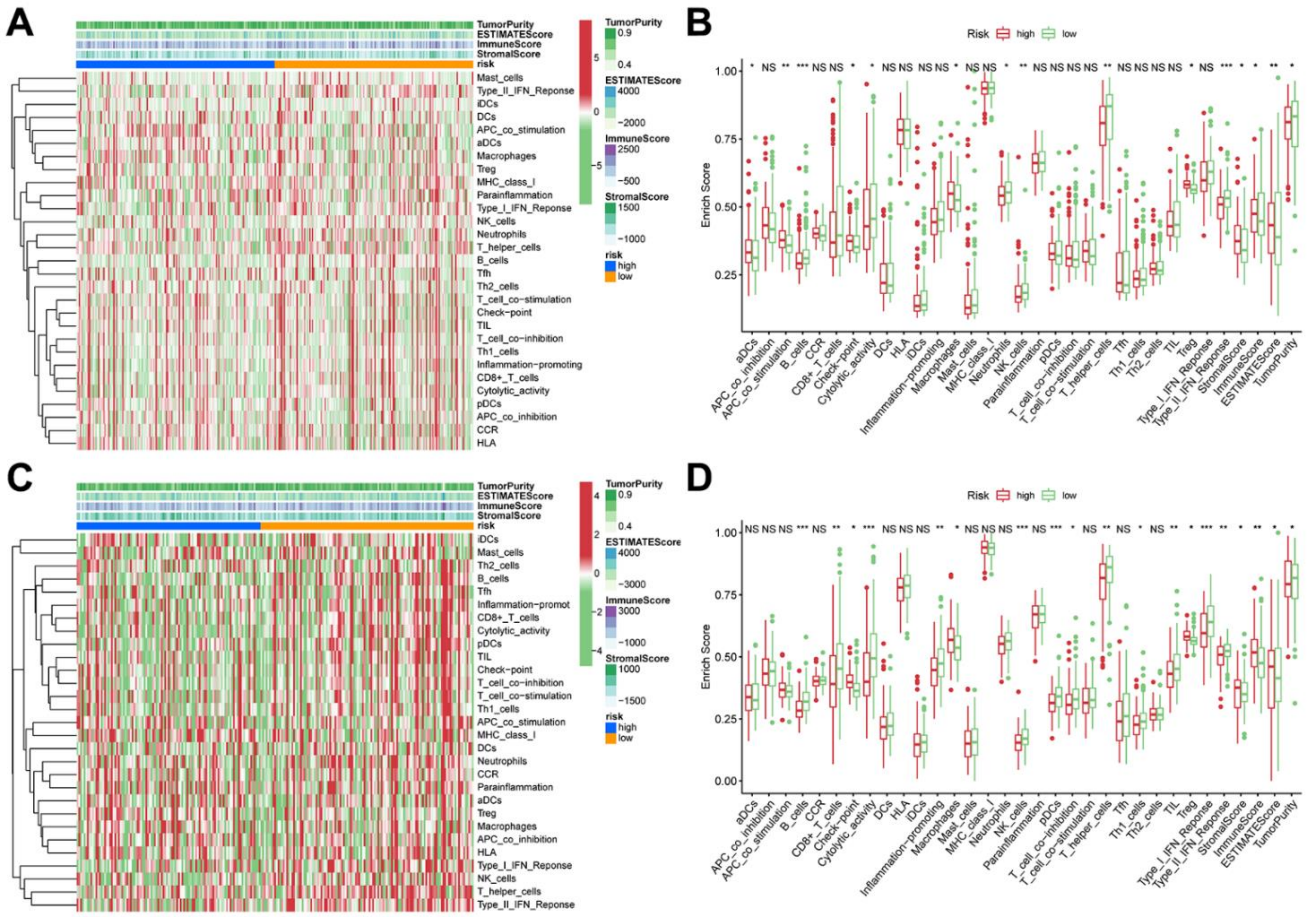
**ssGSEA of immune hallmarks.** (**A**) Heatmap of ssGSEA scores among high- and low-risk groups in training sets (green = negative, red = positive). (**B**) Boxplot of ssGSEA scores, immune score, stromal score, ESTIMATE score, and tumor purity among high- and low-risk groups in TCGA cohorts. (**C**) Heatmap of ssGSEA scores among high- and low-risk groups in validation sets (green = negative, red = positive). (**D**) Boxplot of ssGSEA scores, immune score, stromal score, ESTIMATE score, and tumor purity among high- and low-risk groups in ICGC cohorts. ***p < 0.001, **p < 0.001, *p < 0.05.

TIICs are essential components of the TME. This study examined the relative fraction of 22 immune cell types before computing using the “CIBERSORT” algorithm. The boxplots display the differences in fractions among low and high-risk sets (Figure 5A, 5D). Notably, the high-risk group had substantially higher proportions of M2, M1, and M0 macrophages, and regulatory T cells (Tregs) than the low-risk group. Additionally, M1 macrophages and risk scores had a significant negative correlation (Figure 5B, 5E), whereas M2 macrophages positively correlated to risk scores (Figure 5C, 5F) in TCGA and ICGC cohorts, respectively. Consequently, it is suggested that the risk scores associated with the decreased M1 macrophage and increased M2 macrophages may alter the prognosis of HCC patients. The ssGSEA scores were used to quantify the abundances and activities of various pathways, immunocytes, or functions. Higher ssGSEA scores revealed significant infiltration of immune cells and more active immune-related pathways. The heatmaps (Figure 6A, 6C) and boxplots (Figure 6B, 6D) revealed that the high-risk group had higher ssGSEA scores for various immune cell types, such as checkpoint, macrophages, and Tregs. Furthermore, we evaluated the stromal and immunological scores, which represent the presence of stromal cells and infiltration of immune cells in the tumor microenvironment, respectively. The analysis revealed that samples in the high-risk group exhibited significantly higher immune and stromal scores unlike those in the low-risk set (Figure 6B, 6D). The HCC samples with elevated risk scores exhibited higher levels of infiltrating stromal and immune cells. The TIICs and immune-related pathways were not substantially distinct among high and low-risk groups. In the high-risk group, the levels of immunosuppressive TIICs like M2 and Tregs were substantially higher than the baseline.

### Correlation between immune modulators and risk score

**Figure 7:**
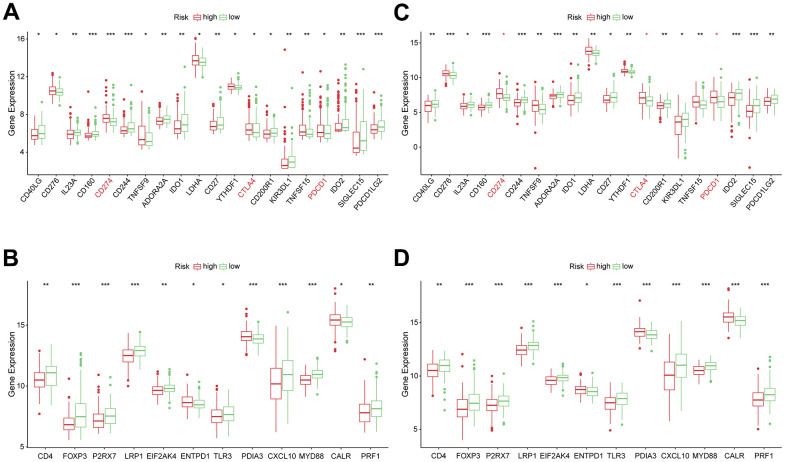
**Correlation between risk subtypes and ICPs and ICD modulators.** (**A**) Differential expression of ICP genes among the risk subtypes in TCGA cohorts. (**B**) Differential expression of ICD modulator genes among the risk subtypes in TCGA cohorts. (**C**) Differential expression of ICP genes among the risk subtypes in ICGC cohorts. (**D**) Differential expression of ICD modulator genes among the risk subtypes in ICGC cohorts.

The levels of genes related to ICD and ICPs were analyzed to explore the potential impact of these modulators on anticancer immunity. All 20 genes, related with ICPs, showed differential expression among the different risk groups in the TCGA and ICGC cohort (Figure 7A, 7C). The expressions of key ICPs including CTLA4, CD274 (PD-L1), and PDCD1 (PD-1) were significantly elevated in the high-risk group. Moreover, both in TCGA and ICGC cohorts, all 12 ICD genes had differential expression among the low and high-risk groups (Figure 7B, 7D). These findings imply that the risk score value, which reflects the expression levels of ICD and ICPs modulators, could be a promising immune treatment biomarker.

### The correlation of risk score with somatic mutation rates and gene set variation analysis

**Figure 8:**
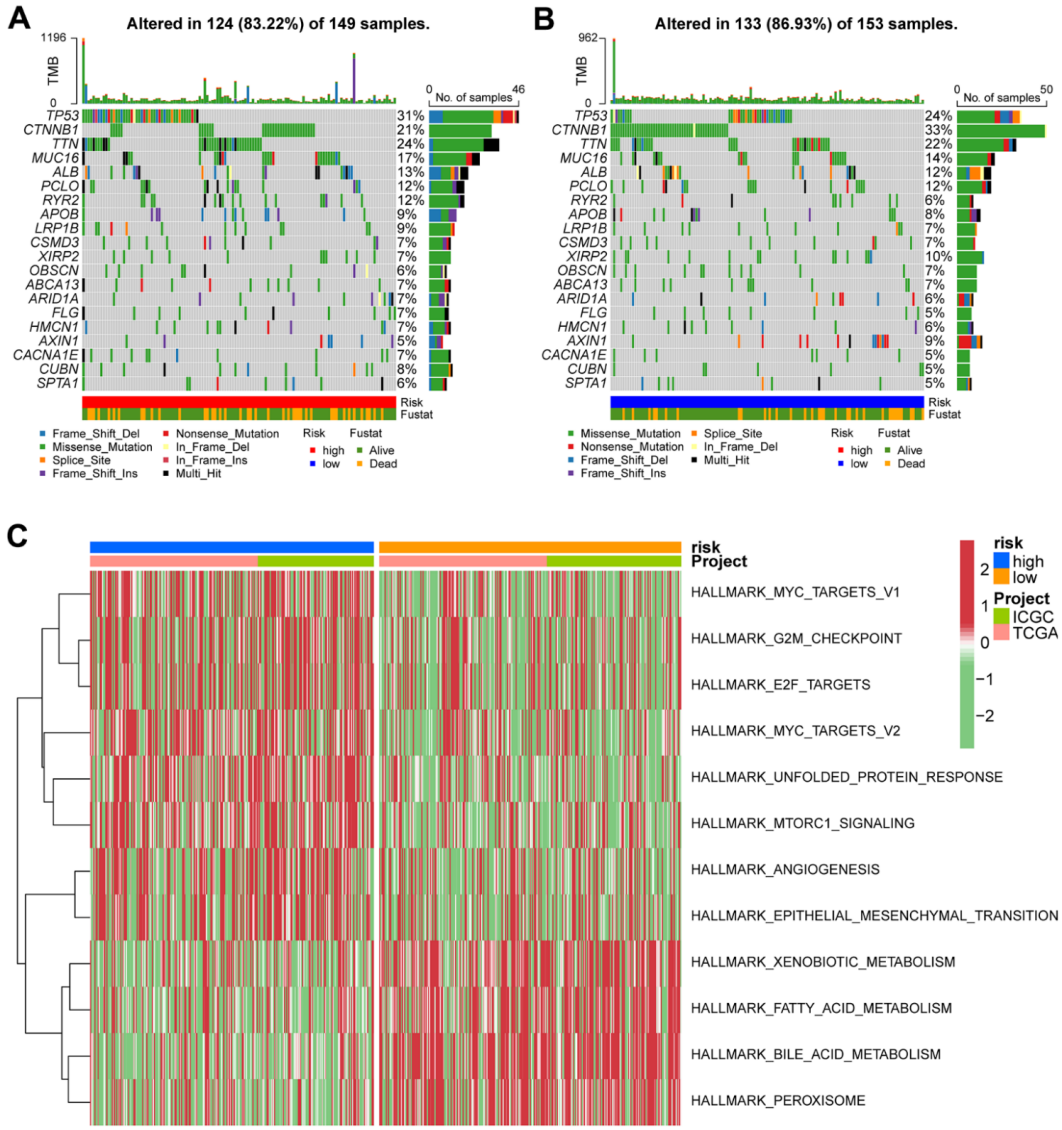
**Gene set variation analysis and correlation between mutation and risk subtype.** (**A**) Top 20 highly mutated genes in HCC high-risk group. (**B**) Top 20 highly mutated genes in HCC low-risk group. (**C**) Heatmap for the contribution of GSVA scores of hallmarks in high- and low-risk groups. The red color represents up-regulated terms in the high-risk group, green color shows the down-regulated terms in the low-risk group.

The mutation landscape of both groups was analyzed. Results showed that the top 20 frequently mutated genes in each subtype included *PCLO, ALB, MUC16, TTN, TP53,* and *CTNNB1* (Figure 8A, 8B). These suggests that the VPRS-based risk score in HCC patients can predict the somatic mutation rate. Moreover, patients with higher risk scores showed good response to anticancer immunity. Using the GSVA, the pathway activities were compared between the low and high-risk groups. Signaling pathways associated with cell cycle, tumor proliferation, tumor invasion, angiogenesis, and tumorigenesis were significantly enriched in the high-risk groups (Figure 8C). The pathways included MYC target v1 and v2, E2F target, G2M checkpoint, MTORC1 signaling, epithelial-mesenchymal transition, and others. Collectively, these findings showed that the VPRS-based risk score can be used as a novel biomarker of HCC, associated with the signaling pathways involved in angiogenesis and tumor invasion.

### Identification of hub genes of vascular endothelial growth factor production-related risk signature

**Figure 9:**
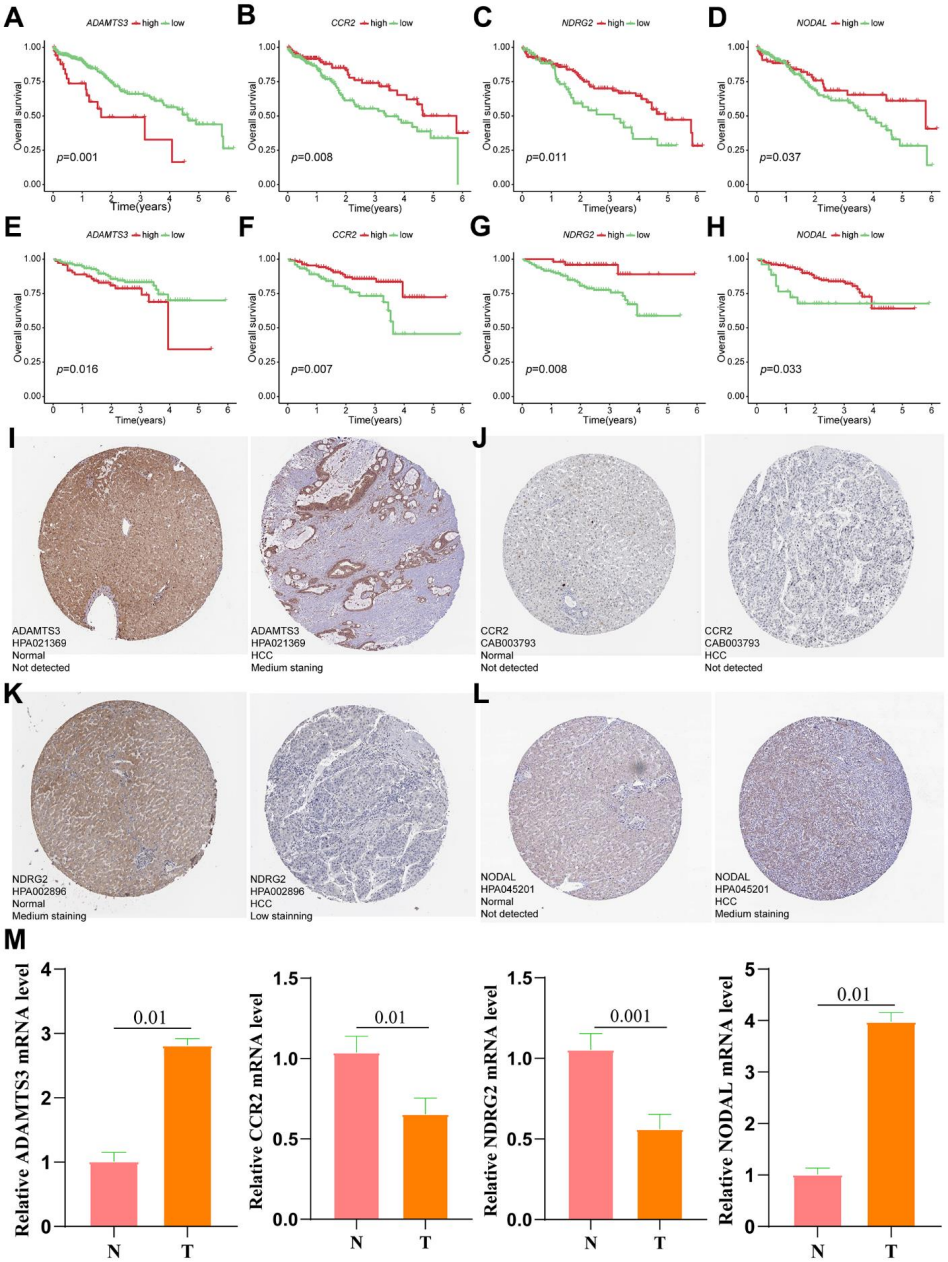
**Verification of the prognostic value and expression of hub genes of VPRS.** Survival analysis of *ADAMTS3*, *CCR2*, *NDRG2*, and *NODAL* for patients in TCGA (**A**–**D**) and ICGC (**E**–**H**) cohorts. The protein expression level of ADAMTS3 (**I**), CCR2 (**J**), NDRG2 (**K**), and NODAL (**L**) in normal and HCC tissues based on the HPA database. (**M**) The relative mRNA expression levels of *ADAMTS3*, *CCR2*, *NDRG2*, and *NODAL* are compared between HCC and non-tumor tissues with real-time PCR results. ***p < 0.001, **p < 0.001, *p < 0.05.

The prognostic value of the 4-gene VPRS was evaluated in the training and validation groups (Figure 9A–9H). Analysis of the effect of the 4 genes on the survival outcomes (Table 2) revealed that *CCR2, NDRG2,* and *NODAL* levels were positively correlated to the OS of HCC patients. On the other hand, those with higher ADAMTS3 levels predicted poor OS. Given the significant correlation between these four genes and the prognosis of HCC patients, *ADAMTS3, CCR2, NDRG2* and *NODAL* were considered as hub genes. Next, we explored the protein expression of related hub genes utilizing the HPA database. Consequently, the expression of *ADAMTS3* (liver#img/ENSG00000156140-ADAMTS3/HPA021369 available from v22.0.proteinatlas.org; liver+cancer#img/ENSG00000156140-ADAMTS3/HPA021369 available from v22.0.proteinatlas.org) and *NODAL* (liver#img/ENSG00000156574-NODAL/HPA045201 available from v22.0.proteinatlas.org; liver+cancer#img/ENSG00000156574-NODAL/HPA045201 available from v22.0.proteinatlas.org) were significantly higher in HCC tissues compared with the normal liver tissues, whereas *NDRG2* (liver#img/ENSG00000165795-NDRG2/HPA002896 available from v22.0.proteinatlas.org; liver+cancer#img/ENSG00000165795-NDRG2/HPA002896 available from v22.0.proteinatlas.org) expression was lower in HCC tissues. The CCR2 protein (liver#img/ENSG00000121807-CCR2/CAB003793 available from v22.0.proteinatlas.org; liver+cancer#img/ENSG00000121807-CCR2/CAB003793 available from v22.0.proteinatlas.org) expression was not detected neither in HCC nor normal tissue (Figure 9I–9L). Furthermore, the expression levels of the four hub genes in our hospital samples were measured using the real-time PCR technique (Figure 9M). Supplementary Table 3 shows the primer sequences of 4 hub genes**.** The mRNA expression of *ADAMTS3, CCR2, NDRG2*, and *NODAL* was significantly higher in HCC than in normal liver tissues, similar to the documented results in the public database.

**Table 2:** 4 VPRS genes related K–M survival analysis in ICGC and TCGA.

**Gene**	**TCGA**		**ICGC**	
	**HR(high)**	**Log-rank *P***	**HR(high)**	**Log-rank *P***
***ADAMTS3***	1.438	*p*<0.05	1.069	*p*<0.05
***CCR2***	0.858	*p*<0.05	0.868	*p*<0.05
***NDRG2***	0.784	*p*<0.05	0.864	*p*<0.05
***NODAL***	0.752	*p*<0.05	0.82	*p*<0.05

### Establishing a prognostic nomogram

**Figure 10:**
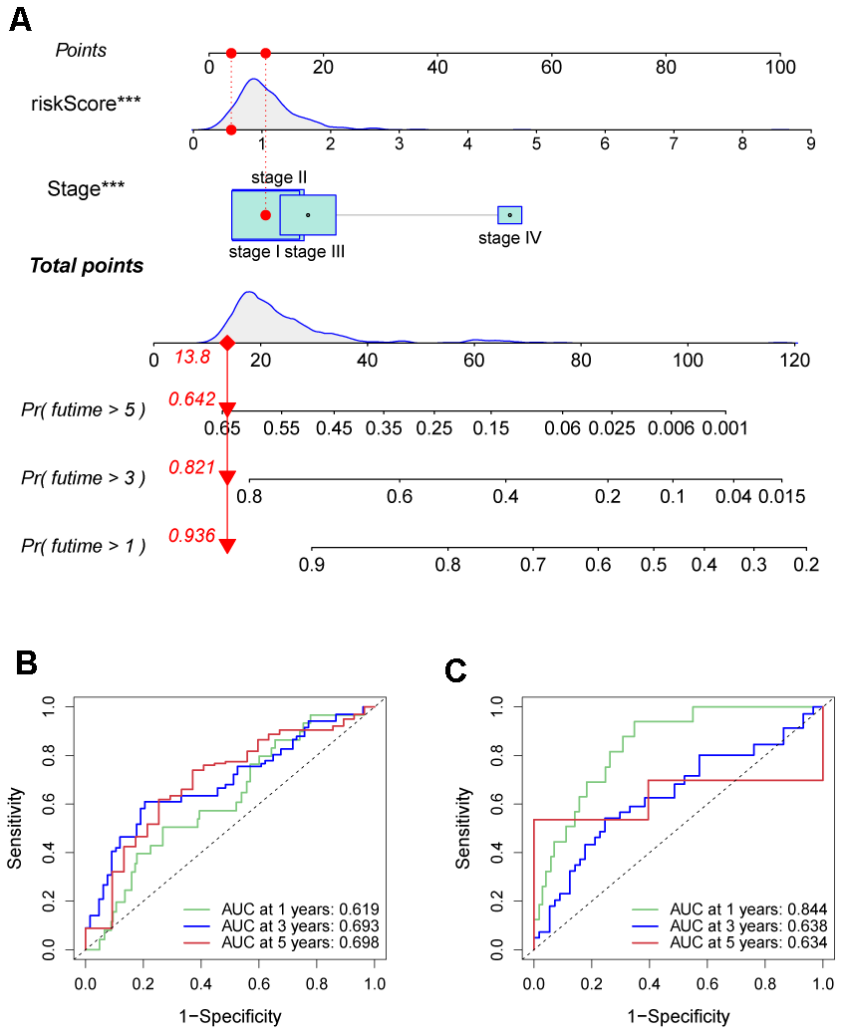
**Prognostic nomogram was established by combining risk score and tumor stage characters.** (**A**) Nomogram for assessing the 1-, 3-, and 5-year OS for HCC patients in the TCGA dataset. ROC curves of 1-, 3-, and 5-year in the TCGA (**B**) and ICGC (**C**) datasets.

A new prognosis nomogram was developed by combining risk score and tumor stage to predict the survival of HCC patients (Figure 10A). The nomogram predicted the OS of patients at 1, 3, and 5 years, with higher scores indicating lower survival probability. The results were comparable with the tumor stage, as higher stage status corresponded to a worse prognosis. To assess the effectiveness of the nomogram in predicting patient survival rates, we performed ROC curve analyses for 1-, 3-, and 5-year survival predictions in both training and validation sets. In the training set, the AUC values indicating the model’s discriminatory power was 0.619, 0.693, and 0.698 for 1-, 3-, and 5-year survival outcomes, respectively (Figure 10B). In the validation set, AUC values were 0.844, 0.638, and 0.634 for the same prediction periods (Figure 10C).

## DISCUSSION

HCC is characterized by high vascularity and degree of malignancy due to impaired angiogenesis [38, 39]. This is because the tumor vascular network determines the supply of oxygen and nutrients to the growing tumor cells [40, 41]. Existing blood vessels are transformed into new ones through a process referred to as angiogenesis [42]. The process of angiogenesis has been shown to modulate cancer progression [43]. Anti-angiogenic therapy is now recognized as an effective strategy for treating cancers. Numerous retrospective studies have shown that microangiogenesis can contribute to the early recurrence and prognosis of HCC [44, 45]. Microangiogenesis levels are often linked to a higher probability of recurrence even poorer survival outcomes [46]. Given the negative prognostic implications of microangiogenesis in recurrent HCC, clinicians should consider whether more aggressive treatment options are necessary for such patients, particularly those experiencing recurrence within two years of their initial curative hepatectomy. This is because HCCs that recur within this time frame are assumed to arise from residual microscopic lesions. However, this issue has not been investigated. Limited research has explored this topic [45]. VEGF functions as an irreplaceable role in the angiogenesis of HCC tissues which is bound up with its tumorigenesis. Although some studies have constructed predictive models of angiogenesis in HCC, none of them has involved VEGF as an important factor in promoting angiogenesis in HCC.

For our study, we first obtained the VPRGs from the Molecular Signatures Database, and found the expression levels of most VPRGs substantially fluctuated among HCC and normal tissues. In addition, these genes had lower CNV and SNP in HCC tissues, implying that these genes are associated with HCC progression. Similarly, it suggested that they had high genetic stability and could inhibit the gene mutations. Through single-factor analysis, we identified prognostic genes in HCC. Using Lasso regression, we built a risk model based on four vascular pattern regulatory genes (VPRGs): *ADAMTS3, CCR2, NDRG2,* and *NODAL*. This model outperformed other clinical factors in predicting patient prognosis significantly. Furthermore, this signature showed better predictive performance for HCC prognosis compared to other clinical factors. A positive correlation was observed between the constructed risk score and the grade, stage, and T stage of the tumor, demonstrating a substantial positive correlation between the risk model we constructed and the HCC progression. Moreover, there was a significant association of our risk score with the clinical characteristic of vascular invasion. Specifically, the risk of significant vascular invasion increases with score, confirming the correlation between our constructed risk model and angiogenesis. We further investigated the validity of our risk model by examining its correlations with clinical features of HCC patients. Through GSVA analysis, the potential mechanisms driving the model’s predictive power were also explored. Furthermore, experiments were conducted to validate the aberrant expression of four key model-related genes identified as VPRGs in HCC tissues. The results showed significant upregulation of *ADAMTS3* and *NODAL*, while *CCR2* and *NDRG2* were downregulated. A Disintegrin and Metalloproteinase with Thrombospondin Motifs 3 (ADAMTS3) is a metalloproteinase, belonging to the ADAMTS family, which is capable of degrading components of the extracellular matrix, like the elastic fibers, collagen and proteoglycans [47]. ADAMTS3 participates in normal physiological processes, like the embryonic development, tissue remodeling and wound healing [48]. A study by Kim et al. reported that *ADAMTS3* was highly expressed in glioma stem cells and significantly correlated with its tumorigenic and proliferative activities [49]. However, at present, no study has reported the correlation between ADAMTS3 and HCC. We found, for the first time, that *ADAMTS3* was highly expressed in HCC samples in our study. Based on these findings, future mechanistic studies should investigate the role of *ADAMTS3* in hepatocarcinogenesis. C-C motif chemokine receptor 2 (CCR2), a C-C chemokine receptor, is mainly expressed on immune cells, like the monocytes, macrophages, and T-cells, and can recognize and bind to chemokines, such as CCL2, as well as modulate the migration of immune cells to inflammatory and tumor sites [50]. In the advanced stage of HCC, CCR2 inhibits anti-tumor immune responses by recruiting and activating immunosuppressive cells, such as the macrophages, myeloid-derived suppressor cells, and regulatory T cells, thereby promoting immune escape of HCC cells [51]. In addition, CCR2 promotes HCC development by modulating fibrosis and inflammatory responses in the liver [52]. In contrast, in the early stages of HCC, immune cells such as macrophages recruited by CCR2 can prevent early tumor formation by eliminating senescent hepatocytes [53]. This intricate observation suggests that CCR2 plays a multifaceted role in HCC progression. It can promote and suppress cancer, which need to be further investigated to clarify the mechanisms governing its divergent effects [54, 55]. *NDRG2*, a tumor suppressor gene, has been shown to inhibit the growth and metastasis of many malignant tumors, including HCC, and its expression level is positively associated with the prognosis and survival outcomes of cancer patients [56–58]. In HCC, the expression of NDRG2 is downregulated, which is consistent with our findings, and down-regulation of NDRG2 expression can significantly increase tumor angiogenesis via the VEGFA pathway [59]. Nodal Growth Differentiation Factor (NODAL) is a secreted protein belonging to the transforming growth factor β (TGF-β) superfamily. It plays a role in physiological processes such as embryonic development, tissue remodeling and stem cell maintenance [60]. However, NODAL are highly expressed in several malignant tumors, such as melanoma, breast cancer, gastric cancer, HCC, etc., and negatively correlates with the degree of differentiation, clinical stage, metastasis, and prognosis of the tumors [61–63]. NODAL is an important component of the TGF-β signaling pathway which enhances the proliferation, migration, and invasion of HCC cells by promoting the phosphorylation of Smad3 and the expression of Snail, drug resistance, and epithelial-mesenchymal transition (EMT) [64]. In addition, NODAL has been shown to modulate tumor cell plasticity by promoting the formation of angiogenic mimetic structures [7]. These results suggest that these signature-associated VPRGs contribute to HCC development and progression, and further studies are advocated to explore their mechanisms and identify potential therapeutic targets.

Immune cells determine response to immunotherapy and tumor survival as the primary component of TIME. For example, clinical research confirms that the immune cell composition of tumors in HCC is strongly associated with patient prognosis and affects therapeutic response [65–67]. Individuals in the low-risk group experienced intense immune cell infiltration compared to their counterparts in the high-risk group. High-density Tumor-Associated Macrophages (TAM) infiltration in HCC is a poor prognosis marker, and TAM is primarily M2 macrophages [68, 69]. M2 macrophages can up-regulate the PDL1 expression in HCC, limiting the activity of CD8+ T cells [70]. In our study, high-risk patients exhibited higher levels of M2 macrophage and Treg cell infiltration, but M1 macrophage infiltration was minimal. Our results strongly demonstrated that the risk scores were negatively correlated with the degree of M1 macrophage infiltration and positively associated with the degree of M2 macrophage infiltration. In our analysis, the high-risk group exhibited higher immune and interstitial scores unlike the low-risk group, although the tumor purity was lower, suggesting better response to immunotherapy. Therefore, boosting anti-tumor immune responses is critical for effective clinical treatment.

Cancer immunotherapy is a new treatment approach for tumors which aims to restart the tumor immune cycle and reestablish the normal anti-tumor immune response [71, 72]. Several studies have investigated the effect of tumor immunotherapy on the TIME [73, 74]. The ICP is an effective approach for stimulating the anti-tumor immune response. The PD-1-and CTLA-4-activated immune T cells are the reliable point for the treatment. Substances that suppress PD-1 and CTLA-4 could significantly enhance the treatment for advanced cancers [75]. Chemokines can also influence cancer migration and progression [76, 77]. Specifically, several key immune checkpoints (e.g., PD-1, CTLA-4) and genes involved in immunogenic cell death pathways (e.g., ERp5, CRTL) were upregulated in the high-risk group. Therefore, higher gene expression levels are more sensitive to ICIs and immunotherapy. Thus, our risk-scoring model is expected to improve individualized treatment of HCC patients.

The intricacy of HCC pathogenesis pathways leads to variation across diverse populations in various countries and regions, posing a significant challenge to customized therapy [78, 79]. Therefore, the use of a single DEG as a biomarker for customized HCC patients is not reliable. Moreover, it is difficult to identify and characterize the immune properties of HCC. In our study, we developed a new prognostic biomarker of HCC that can differentiate the immune status and the malignancy of HCC as well as predict the efficacy of immune checkpoint blockade (ICB) treatment in HCC patients. In addition, a microangiogenesis prediction model for HCC prognosis was developed and validated. Nevertheless, we acknowledge that our study has certain limitations. Firstly, the retrospective nature of our study, although validated with diverse datasets to assess performance, limits the application of our model. Secondly, the TIME exhibited significant heterogeneity, making it difficult to perform precise evaluation. Despite efforts to minimize biases by leveraging the relative order of immune cell infiltration, residual biases may persist. Future biological analyses are advocated to expand our understanding of survival outcomes.

## Supplementary Material

Supplementary Figures

Supplementary Table 1

Supplementary Tables 2 and 3

## References

[1] Wang Y, Song F, Zhang X, Yang C. Mitochondrial-Related Transcriptome Feature Correlates with Prognosis, Vascular Invasion, Tumor Microenvironment, and Treatment Response in Hepatocellular Carcinoma. Oxid Med Cell Longev. 2022; 2022:1592905. 10.1155/2022/1592905 35535359 PMC9078845

[2] Akinyemiju T, Abera S, Ahmed M, Alam N, Alemayohu MA, Allen C, Al-Raddadi R, Alvis-Guzman N, Amoako Y, Artaman A, Ayele TA, Barac A, Bensenor I, et al, and Global Burden of Disease Liver Cancer Collaboration. The Burden of Primary Liver Cancer and Underlying Etiologies From 1990 to 2015 at the Global, Regional, and National Level: Results From the Global Burden of Disease Study 2015. JAMA Oncol. 2017; 3:1683–91. 10.1001/jamaoncol.2017.3055 28983565 PMC5824275

[3] Morse MA, Sun W, Kim R, He AR, Abada PB, Mynderse M, Finn RS. The Role of Angiogenesis in Hepatocellular Carcinoma. Clin Cancer Res. 2019; 25:912–20. 10.1158/1078-0432.CCR-18-1254 30274981

[4] Felmeden DC, Blann AD, Lip GY. Angiogenesis: basic pathophysiology and implications for disease. Eur Heart J. 2003; 24:586–603. 10.1016/s0195-668x(02)00635-8 12657217

[5] Karamysheva AF. Mechanisms of angiogenesis. Biochemistry (Mosc). 2008; 73:751–62. 10.1134/s0006297908070031 18707583

[6] Jiang X, Wang J, Deng X, Xiong F, Zhang S, Gong Z, Li X, Cao K, Deng H, He Y, Liao Q, Xiang B, Zhou M, et al. The role of microenvironment in tumor angiogenesis. J Exp Clin Cancer Res. 2020; 39:204. 10.1186/s13046-020-01709-5 32993787 PMC7526376

[7] Lugano R, Ramachandran M, Dimberg A. Tumor angiogenesis: causes, consequences, challenges and opportunities. Cell Mol Life Sci. 2020; 77:1745–70. 10.1007/s00018-019-03351-7 31690961 PMC7190605

[8] Pinto E, Pelizzaro F, Farinati F, Russo FP. Angiogenesis and Hepatocellular Carcinoma: From Molecular Mechanisms to Systemic Therapies. Medicina (Kaunas). 2023; 59:1115. 10.3390/medicina59061115 37374319 PMC10305396

[9] Hoang MV, Nagy JA, Fox JE, Senger DR. Moderation of calpain activity promotes neovascular integration and lumen formation during VEGF-induced pathological angiogenesis. PLoS One. 2010; 5:e13612. 10.1371/journal.pone.0013612 21049044 PMC2963609

[10] Bates DO. Vascular endothelial growth factors and vascular permeability. Cardiovasc Res. 2010; 87:262–71. 10.1093/cvr/cvq105 20400620 PMC2895541

[11] Chen L, Lin G, Chen K, Liang R, Wan F, Zhang C, Tian G, Zhu X. VEGF promotes migration and invasion by regulating EMT and MMPs in nasopharyngeal carcinoma. J Cancer. 2020; 11:7291–301. 10.7150/jca.46429 33193893 PMC7646165

[12] Florentin J, O’Neil SP, Ohayon LL, Uddin A, Vasamsetti SB, Arunkumar A, Ghosh S, Boatz JC, Sui J, Kliment CR, Chan SY, Dutta P. VEGF Receptor 1 Promotes Hypoxia-Induced Hematopoietic Progenitor Proliferation and Differentiation. Front Immunol. 2022; 13:882484. 10.3389/fimmu.2022.882484 35634304 PMC9133347

[13] Farzaneh Z, Vosough M, Agarwal T, Farzaneh M. Critical signaling pathways governing hepatocellular carcinoma behavior; small molecule-based approaches. Cancer Cell Int. 2021; 21:208. 10.1186/s12935-021-01924-w 33849569 PMC8045321

[14] Zhao P, Malik S, Xing S. Epigenetic Mechanisms Involved in HCV-Induced Hepatocellular Carcinoma (HCC). Front Oncol. 2021; 11:677926. 10.3389/fonc.2021.677926 34336665 PMC8320331

[15] Xu C, Xu Z, Zhang Y, Evert M, Calvisi DF, Chen X. β-Catenin signaling in hepatocellular carcinoma. J Clin Invest. 2022; 132:e154515. 10.1172/JCI154515 35166233 PMC8843739

[16] Haibe Y, Kreidieh M, El Hajj H, Khalifeh I, Mukherji D, Temraz S, Shamseddine A. Resistance Mechanisms to Anti-angiogenic Therapies in Cancer. Front Oncol. 2020; 10:221. 10.3389/fonc.2020.00221 32175278 PMC7056882

[17] Cai C, Wang X, Fu Q, Chen A. The VEGF expression associated with prognosis in patients with intrahepatic cholangiocarcinoma: a systematic review and meta-analysis. World J Surg Oncol. 2022; 20:40. 10.1186/s12957-022-02511-7 35189920 PMC8859901

[18] Khodabakhsh F, Merikhian P, Eisavand MR, Farahmand L. Crosstalk between MUC1 and VEGF in angiogenesis and metastasis: a review highlighting roles of the MUC1 with an emphasis on metastatic and angiogenic signaling. Cancer Cell Int. 2021; 21:200. 10.1186/s12935-021-01899-8 33836774 PMC8033681

[19] Arciero CA, Sigurdson ER. Liver-directed therapies for hepatocellular carcinoma. J Natl Compr Canc Netw. 2006; 4:768–74. 10.6004/jnccn.2006.0067 16948955

[20] Jácome AA, Castro AC, Vasconcelos JP, Silva MH, Lessa MA, Moraes ED, Andrade AC, Lima FM, Farias JP, Gil RA, Prolla G, Garicochea B. Efficacy and Safety Associated With Immune Checkpoint Inhibitors in Unresectable Hepatocellular Carcinoma: A Meta-analysis. JAMA Netw Open. 2021; 4:e2136128. 10.1001/jamanetworkopen.2021.36128 34870682 PMC8649834

[21] Syed YY. Ramucirumab: A Review in Hepatocellular Carcinoma. Drugs. 2020; 80:315–22. 10.1007/s40265-020-01263-6 32034692

[22] Liu Y, Li Y, Wang Y, Lin C, Zhang D, Chen J, Ouyang L, Wu F, Zhang J, Chen L. Recent progress on vascular endothelial growth factor receptor inhibitors with dual targeting capabilities for tumor therapy. J Hematol Oncol. 2022; 15:89. 10.1186/s13045-022-01310-7 35799213 PMC9263050

[23] Nuti M, Zizzari IG, Botticelli A, Rughetti A, Marchetti P. The ambitious role of anti angiogenesis molecules: Turning a cold tumor into a hot one. Cancer Treat Rev. 2018; 70:41–46. 10.1016/j.ctrv.2018.07.016 30077081

[24] Jiang X, Xu Y, Chen D, Wang M, Qiu M, Xiong L, Zhang L, Yu H, Xiong Z. A Novel Angiogenesis-Related Prognostic Signature Associated with the Hepatocellular Carcinoma Immune Microenvironment and Survival Outcome. Int J Gen Med. 2022; 15:311–23. 10.2147/IJGM.S349210 35027841 PMC8752972

[25] Ci H, Wang X, Shen K, Du W, Zhou J, Fu Y, Dong Q, Jia H. An Angiogenic Gene Signature for Prediction of the Prognosis and Therapeutic Responses of Hepatocellular Carcinoma. Int J Mol Sci. 2023; 24:3324. 10.3390/ijms24043324 36834736 PMC9965274

[26] Yang Y, Wu G, Li Q, Zheng Y, Liu M, Zhou L, Chen Z, Wang Y, Guo Q, Ji R, Zhou Y. Angiogenesis-Related Immune Signatures Correlate With Prognosis, Tumor Microenvironment, and Therapeutic Sensitivity in Hepatocellular Carcinoma. Front Mol Biosci. 2021; 8:690206. 10.3389/fmolb.2021.690206 34262941 PMC8273615

[27] Zhen Z, Shen Z, Hu Y, Sun P. Screening and identification of angiogenesis-related genes as potential novel prognostic biomarkers of hepatocellular carcinoma through bioinformatics analysis. Aging (Albany NY). 2021; 13:17707–33. 10.18632/aging.203260 34252885 PMC8312452

[28] Ansari MJ, Bokov D, Markov A, Jalil AT, Shalaby MN, Suksatan W, Chupradit S, Al-Ghamdi HS, Shomali N, Zamani A, Mohammadi A, Dadashpour M. Cancer combination therapies by angiogenesis inhibitors; a comprehensive review. Cell Commun Signal. 2022; 20:49. 10.1186/s12964-022-00838-y 35392964 PMC8991477

[29] Bolstad BM, Irizarry RA, Astrand M, Speed TP. A comparison of normalization methods for high density oligonucleotide array data based on variance and bias. Bioinformatics. 2003; 19:185–93. 10.1093/bioinformatics/19.2.185 12538238

[30] Ritchie ME, Phipson B, Wu D, Hu Y, Law CW, Shi W, Smyth GK. limma powers differential expression analyses for RNA-sequencing and microarray studies. Nucleic Acids Res. 2015; 43:e47. 10.1093/nar/gkv007 25605792 PMC4402510

[31] Miller RG, Jackson CE, Kasarskis EJ, England JD, Forshew D, Johnston W, Kalra S, Katz JS, Mitsumoto H, Rosenfeld J, Shoesmith C, Strong MJ, Woolley SC, and Quality Standards Subcommittee of the American Academy of Neurology. Practice parameter update: the care of the patient with amyotrophic lateral sclerosis: multidisciplinary care, symptom management, and cognitive/behavioral impairment (an evidence-based review): report of the Quality Standards Subcommittee of the American Academy of Neurology. Neurology. 2009; 73:1227–33. 10.1212/WNL.0b013e3181bc01a4 19822873 PMC2764728

[32] Swets JA. Measuring the accuracy of diagnostic systems. Science. 1988; 240:1285–93. 10.1126/science.3287615 3287615

[33] Ciardullo C, Aptullahoglu E, Woodhouse L, Lin WY, Wallis JP, Marr H, Marshall S, Bown N, Willmore E, Lunec J. Non-genotoxic MDM2 inhibition selectively induces a pro-apoptotic p53 gene signature in chronic lymphocytic leukemia cells. Haematologica. 2019; 104:2429–42. 10.3324/haematol.2018.206631 31004033 PMC6959162

[34] Bindea G, Mlecnik B, Tosolini M, Kirilovsky A, Waldner M, Obenauf AC, Angell H, Fredriksen T, Lafontaine L, Berger A, Bruneval P, Fridman WH, Becker C, et al. Spatiotemporal dynamics of intratumoral immune cells reveal the immune landscape in human cancer. Immunity. 2013; 39:782–95. 10.1016/j.immuni.2013.10.003 24138885

[35] Finotello F, Mayer C, Plattner C, Laschober G, Rieder D, Hackl H, Krogsdam A, Loncova Z, Posch W, Wilflingseder D, Sopper S, Ijsselsteijn M, Brouwer TP, et al. Molecular and pharmacological modulators of the tumor immune contexture revealed by deconvolution of RNA-seq data. Genome Med. 2019; 11:34. 10.1186/s13073-019-0638-6 31126321 PMC6534875

[36] Hänzelmann S, Castelo R, Guinney J. GSVA: gene set variation analysis for microarray and RNA-seq data. BMC Bioinformatics. 2013; 14:7. 10.1186/1471-2105-14-7 23323831 PMC3618321

[37] Xu FQ, Dong MM, Wang ZF, Cao LD. Metabolic rearrangements and intratumoral heterogeneity for immune response in hepatocellular carcinoma. Front Immunol. 2023; 14:1083069. 10.3389/fimmu.2023.1083069 36776894 PMC9908004

[38] Fang JH, Zhou HC, Zhang C, Shang LR, Zhang L, Xu J, Zheng L, Yuan Y, Guo RP, Jia WH, Yun JP, Chen MS, Zhang Y, Zhuang SM. A novel vascular pattern promotes metastasis of hepatocellular carcinoma in an epithelial-mesenchymal transition-independent manner. Hepatology. 2015; 62:452–65. 10.1002/hep.27760 25711742

[39] Zheng N, Zhang S, Wu W, Zhang N, Wang J. Regulatory mechanisms and therapeutic targeting of vasculogenic mimicry in hepatocellular carcinoma. Pharmacol Res. 2021; 166:105507. 10.1016/j.phrs.2021.105507 33610718

[40] Yang S, Yang C, Yu F, Ding W, Hu Y, Cheng F, Zhang F, Guan B, Wang X, Lu L, Rao J. Endoplasmic reticulum resident oxidase ERO1-Lalpha promotes hepatocellular carcinoma metastasis and angiogenesis through the S1PR1/STAT3/VEGF-A pathway. Cell Death Dis. 2018; 9:1105. 10.1038/s41419-018-1134-4 30377291 PMC6207574

[41] Xue X, Gao W, Sun B, Xu Y, Han B, Wang F, Zhang Y, Sun J, Wei J, Lu Z, Zhu Y, Sato Y, Sekido Y, et al. Vasohibin 2 is transcriptionally activated and promotes angiogenesis in hepatocellular carcinoma. Oncogene. 2013; 32:1724–34. 10.1038/onc.2012.177 22614011

[42] Ribatti D, Djonov V. Intussusceptive microvascular growth in tumors. Cancer Lett. 2012; 316:126–31. 10.1016/j.canlet.2011.10.040 22197620

[43] Lin J, Cao S, Wang Y, Hu Y, Liu H, Li J, Chen J, Li P, Liu J, Wang Q, Zheng L. Long non-coding RNA UBE2CP3 enhances HCC cell secretion of VEGFA and promotes angiogenesis by activating ERK1/2/HIF-1α/VEGFA signalling in hepatocellular carcinoma. J Exp Clin Cancer Res. 2018; 37:113. 10.1186/s13046-018-0727-1 29866133 PMC5987644

[44] Lim KC, Chow PK, Allen JC, Chia GS, Lim M, Cheow PC, Chung AY, Ooi LL, Tan SB. Microvascular invasion is a better predictor of tumor recurrence and overall survival following surgical resection for hepatocellular carcinoma compared to the Milan criteria. Ann Surg. 2011; 254:108–13. 10.1097/SLA.0b013e31821ad884 21527845

[45] Erstad DJ, Tanabe KK. Prognostic and Therapeutic Implications of Microvascular Invasion in Hepatocellular Carcinoma. Ann Surg Oncol. 2019; 26:1474–93. 10.1245/s10434-019-07227-9 30788629

[46] Sun X, Yang Z, Mei J, Lyu N, Lai J, Chen M, Zhao M. The guiding value of microvascular invasion for treating early recurrent small hepatocellular carcinoma. Int J Hyperthermia. 2021; 38:931–38. 10.1080/02656736.2021.1937715 34121576

[47] Li T, Peng J, Li Q, Shu Y, Zhu P, Hao L. The Mechanism and Role of ADAMTS Protein Family in Osteoarthritis. Biomolecules. 2022; 12:959. 10.3390/biom12070959 35883515 PMC9313267

[48] Kumar S, Rao N, Ge R. Emerging Roles of ADAMTSs in Angiogenesis and Cancer. Cancers (Basel). 2012; 4:1252–99. 10.3390/cancers4041252 24213506 PMC3712723

[49] Kim HJ, Jeong HY, Batara DC, Moon C, Lee S, Lee SJ, Park SI, Choi MC, Kim SH. Downregulation of ADAMTS3 Suppresses Stemness and Tumorigenicity in Glioma Stem Cell. CNS Neurosci Ther. 2023; 29:682–90. 10.1111/cns.14052 36514188 PMC9873505

[50] She S, Ren L, Chen P, Wang M, Chen D, Wang Y, Chen H. Functional Roles of Chemokine Receptor CCR2 and Its Ligands in Liver Disease. Front Immunol. 2022; 13:812431. 10.3389/fimmu.2022.812431 35281057 PMC8913720

[51] Fein MR, He XY, Almeida AS, Bružas E, Pommier A, Yan R, Eberhardt A, Fearon DT, Van Aelst L, Wilkinson JE, Dos Santos CO, Egeblad M. Cancer cell CCR2 orchestrates suppression of the adaptive immune response. J Exp Med. 2020; 217:e20181551. 10.1084/jem.20181551 32667673 PMC7537399

[52] Hammerich L, Tacke F. Hepatic inflammatory responses in liver fibrosis. Nat Rev Gastroenterol Hepatol. 2023; 20:633–46. 10.1038/s41575-023-00807-x 37400694

[53] Bartneck M, Schrammen PL, Möckel D, Govaere O, Liepelt A, Krenkel O, Ergen C, McCain MV, Eulberg D, Luedde T, Trautwein C, Kiessling F, Reeves H, et al. The CCR2+ Macrophage Subset Promotes Pathogenic Angiogenesis for Tumor Vascularization in Fibrotic Livers. Cell Mol Gastroenterol Hepatol. 2019; 7:371–90. 10.1016/j.jcmgh.2018.10.007 30704985 PMC6357791

[54] Li X, Wu X, Luo P, Xiong L. Astrocyte-specific NDRG2 gene: functions in the brain and neurological diseases. Cell Mol Life Sci. 2020; 77:2461–72. 10.1007/s00018-019-03406-9 31834421 PMC11104915

[55] Wang J, Liu M, Hou W, Hou M, Zhang L, Sun M, Liu S, Yang H, Guo H, Zhang X, Xie F, Liu Y, Ma Y. N-myc Downstream-Regulated Gene 2 (Ndrg2): A Critical Mediator of Estrogen-Induced Neuroprotection Against Cerebral Ischemic Injury. Mol Neurobiol. 2022; 59:4793–804. 10.1007/s12035-022-02877-5 35622273

[56] Kloten V, Schlensog M, Eschenbruch J, Gasthaus J, Tiedemann J, Mijnes J, Heide T, Braunschweig T, Knüchel R, Dahl E. Abundant NDRG2 Expression Is Associated with Aggressiveness and Unfavorable Patients’ Outcome in Basal-Like Breast Cancer. PLoS One. 2016; 11:e0159073. 10.1371/journal.pone.0159073 27400234 PMC4939972

[57] Li SJ, Wang WY, Li B, Chen B, Zhang B, Wang X, Chen CS, Zhao QC, Shi H, Yao L. Expression of NDRG2 in human lung cancer and its correlation with prognosis. Med Oncol. 2013; 30:421. 10.1007/s12032-012-0421-7 23307246 PMC3586402

[58] Lee DC, Kang YK, Kim WH, Jang YJ, Kim DJ, Park IY, Sohn BH, Sohn HA, Lee HG, Lim JS, Kim JW, Song EY, Kim DM, et al. Functional and clinical evidence for NDRG2 as a candidate suppressor of liver cancer metastasis. Cancer Res. 2008; 68:4210–20. 10.1158/0008-5472.CAN-07-5040 18519680

[59] Wang J, Li T, Ma L, Liu G, Wang G, Kang J. NDRG2 inhibition facilitates angiogenesis of hepatocellular carcinoma. Open Med (Wars). 2021; 16:742–48. 10.1515/med-2021-0268 34013046 PMC8114951

[60] Jia S, Meng A. TGFβ family signaling and development. Development. 2021; 148:dev188490. 10.1242/dev.188490 33712443

[61] Wu Y, Du K, Guan W, Wu D, Tang H, Wang N, Qi J, Gu Z, Yang J, Ding J. A novel definition of microvessel density in renal cell carcinoma: Angiogenesis plus vasculogenic mimicry. Oncol Lett. 2020; 20:192. 10.3892/ol.2020.12054 32952661 PMC7479517

[62] Hong X, Wen B, Zhang H, Li Y, Wu H, Zhao W, Luo X. Biological effects of NODAL on endometrial cancer cells and its underlying mechanisms. Exp Ther Med. 2021; 21:402. 10.3892/etm.2021.9833 33717261 PMC7938447

[63] Kong B, Wang W, Esposito I, Friess H, Michalski CW, Kleeff J. Increased expression of Nodal correlates with reduced patient survival in pancreatic cancer. Pancreatology. 2015; 15:156–61. 10.1016/j.pan.2015.02.001 25708930

[64] Sun C, Sun L, Jiang K, Gao DM, Kang XN, Wang C, Zhang S, Huang S, Qin X, Li Y, Liu YK. NANOG promotes liver cancer cell invasion by inducing epithelial-mesenchymal transition through NODAL/SMAD3 signaling pathway. Int J Biochem Cell Biol. 2013; 45:1099–108. 10.1016/j.biocel.2013.02.017 23474366

[65] Giraud J, Chalopin D, Blanc JF, Saleh M. Hepatocellular Carcinoma Immune Landscape and the Potential of Immunotherapies. Front Immunol. 2021; 12:655697. 10.3389/fimmu.2021.655697 33815418 PMC8012774

[66] Oura K, Morishita A, Tani J, Masaki T. Tumor Immune Microenvironment and Immunosuppressive Therapy in Hepatocellular Carcinoma: A Review. Int J Mol Sci. 2021; 22:5801. 10.3390/ijms22115801 34071550 PMC8198390

[67] Ma J, Kuang L, Zhao R. Establishing a signature based on immunogenic cell death-related gene pairs to predict immunotherapy and survival outcomes of patients with hepatocellular carcinoma. Aging (Albany NY). 2022; 14:9699–714. 10.18632/aging.204419 36516498 PMC9792212

[68] Arvanitakis K, Koletsa T, Mitroulis I, Germanidis G. Tumor-Associated Macrophages in Hepatocellular Carcinoma Pathogenesis, Prognosis and Therapy. Cancers (Basel). 2022; 14:226. 10.3390/cancers14010226 35008390 PMC8749970

[69] Yeung OW, Lo CM, Ling CC, Qi X, Geng W, Li CX, Ng KT, Forbes SJ, Guan XY, Poon RT, Fan ST, Man K. Alternatively activated (M2) macrophages promote tumour growth and invasiveness in hepatocellular carcinoma. J Hepatol. 2015; 62:607–16. 10.1016/j.jhep.2014.10.029 25450711

[70] Ho DW, Tsui YM, Chan LK, Sze KM, Zhang X, Cheu JW, Chiu YT, Lee JM, Chan AC, Cheung ET, Yau DT, Chia NH, Lo IL, et al. Single-cell RNA sequencing shows the immunosuppressive landscape and tumor heterogeneity of HBV-associated hepatocellular carcinoma. Nat Commun. 2021; 12:3684. 10.1038/s41467-021-24010-1 34140495 PMC8211687

[71] Wesch D, Kabelitz D, Oberg HH. Tumor resistance mechanisms and their consequences on γδ T cell activation. Immunol Rev. 2020; 298:84–98. 10.1111/imr.12925 33048357

[72] Zhang X, Xie J, He D, Yan X, Chen J. Cell Pair Algorithm-Based Immune Infiltrating Cell Signature for Improving Outcomes and Treatment Responses in Patients with Hepatocellular Carcinoma. Cells. 2023; 12:202. 10.3390/cells12010202 36611994 PMC9818873

[73] He Q, Yang J, Jin Y. Development and Validation of TACE Refractoriness-Related Diagnostic and Prognostic Scores and Characterization of Tumor Microenvironment Infiltration in Hepatocellular Carcinoma. Front Immunol. 2022; 13:869993. 10.3389/fimmu.2022.869993 35493518 PMC9043752

[74] Hinshaw DC, Shevde LA. The Tumor Microenvironment Innately Modulates Cancer Progression. Cancer Res. 2019; 79:4557–66. 10.1158/0008-5472.CAN-18-3962 31350295 PMC6744958

[75] Rotte A. Combination of CTLA-4 and PD-1 blockers for treatment of cancer. J Exp Clin Cancer Res. 2019; 38:255. 10.1186/s13046-019-1259-z 31196207 PMC6567914

[76] Caronni N, Savino B, Recordati C, Villa A, Locati M, Bonecchi R. Cancer and Chemokines. Methods Mol Biol. 2016; 1393:87–96. 10.1007/978-1-4939-3338-9_8 27033218

[77] Wang T, Chen B, Meng T, Liu Z, Wu W. Identification and immunoprofiling of key prognostic genes in the tumor microenvironment of hepatocellular carcinoma. Bioengineered. 2021; 12:1555–75. 10.1080/21655979.2021.1918538 33955820 PMC8806269

[78] Zhang Y, Xu J, Zhang N, Chen M, Wang H, Zhu D. Targeting the tumour immune microenvironment for cancer therapy in human gastrointestinal malignancies. Cancer Lett. 2019; 458:123–35. 10.1016/j.canlet.2019.05.017 31121212

[79] Wang X, Wu Y, Wen D, Wu LY, Zhao Y, He Y, Yang H. An Individualized Immune Prognostic Index is a Superior Predictor of Survival of Hepatocellular Carcinoma. Med Sci Monit. 2020; 26:e921786. 10.12659/MSM.921786 32527991 PMC7285951

