# Supplementary material for: Identification of VEGFs-related gene signature for predicting microangiogenesis and hepatocellular carcinoma prognosis
Source: Aging (Albany NY). 2024 Jun 13;16(12):10321–47. doi: 10.18632/aging.205931 (PMC11236318; doi:10.18632/aging.205931)
Supplement: Supplementary Figures [file aging-16-205931-s001.pdf]

SUPPLEMENTARY FIGURES

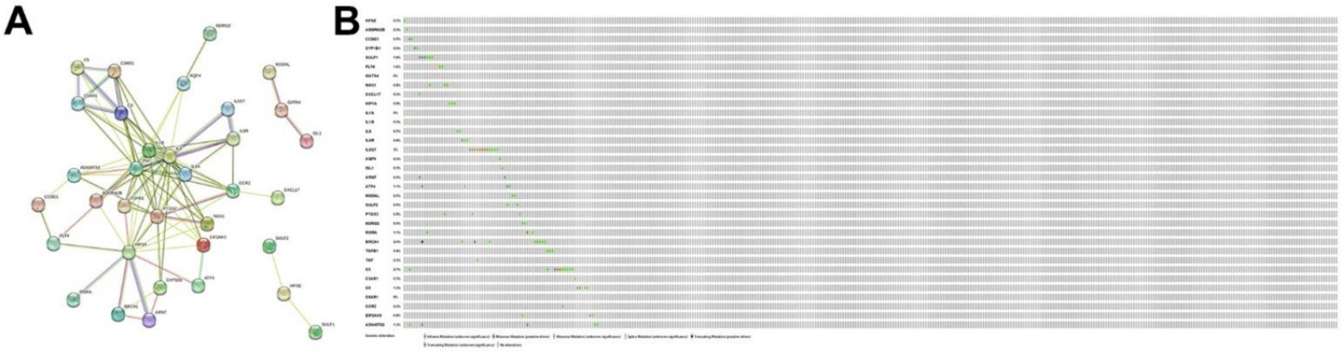

Supplementary Figure 1. The relationships and characters of VEGF production-related genes (VPRGs). (**A**) The PPI network of VPRGs in STRING database. (**B**) The copy number variation and somatic mutational status of GSRGs.

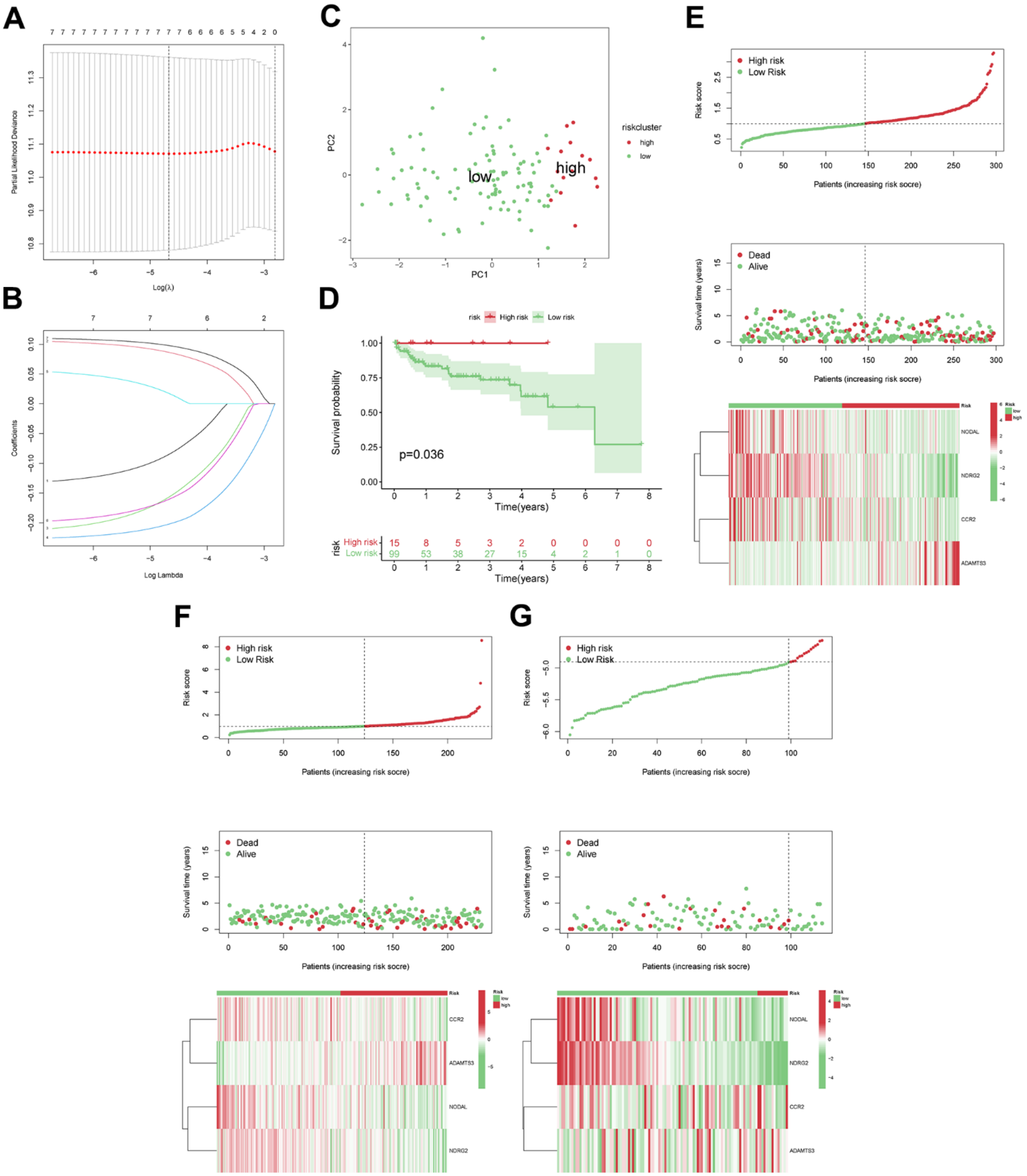

Supplementary Figure 2. LASSO regression analysis. (**A**) Partial likelihood deviance as a function of regularization parameter λ in the training dataset. Each red point marks a λ value along regularization paths, and gray error bars represent confidence intervals for the cross-validated error rate. The left vertical dotted line indicates the minimum error, whereas the right vertical dotted line represents the most significant λ value, the error of which is within 1 SD of the minimum. The horizontal row of numbers above the plot shows the gene number in each condition upon shrinkage and selection as determined by the linear regression. (**B**) The craft plot for partial likelihood deviance in LASSO, different colors represent different genes associated with the VEGF production-related signature (VPRS). (**C**) PCA of HCC samples from the TCGA; dots in red and green represent samples in high-risk and low-risk groups, respectively. (**D**) Overall survival analysis of the risk score of HCC patients in TCGA. The distribution of risk score, corresponding OS, and gene expression based on the TCGA (**E**), ICGC (**F**), and GSE76247 (**G**) sets.

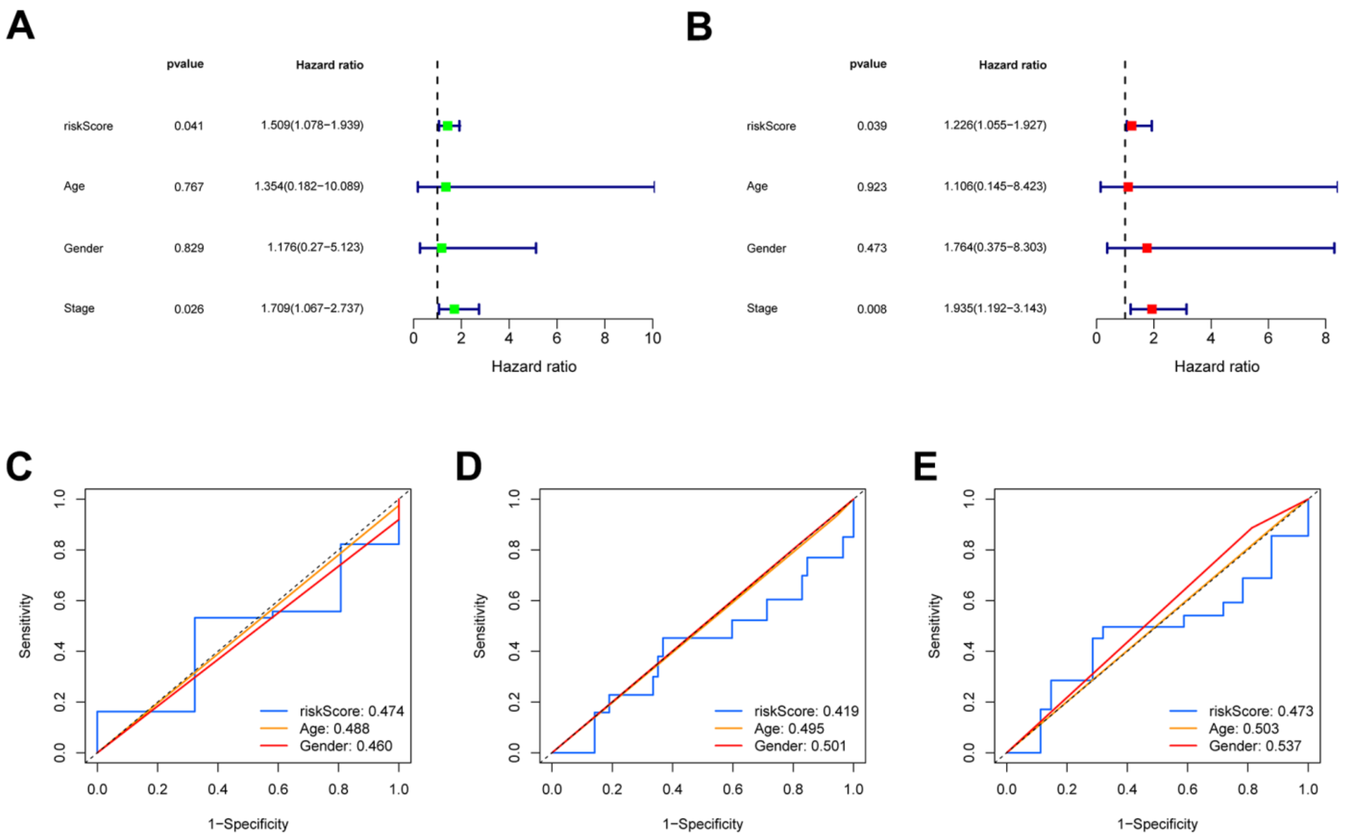

Supplementary Figure 3. The prognostic value of VPRS. In the GSE76427, forest plot on the left for the univariate Cox test (**A**) displaying the correlation of the risk score and clinical factors with patient OS, and forest plot on the right for the multivariate Cox analysis (**B**) used to identify independent risk factors associated with the OS. The ROC curve of clinical factors and risk score for predicting the 1- (**C**), 3- (**D**), and 5-year (**E**) OS.
