# Supplementary material for: Identification of VEGFs-related gene signature for predicting microangiogenesis and hepatocellular carcinoma prognosis
Source: Aging (Albany NY). 2024 Jun 13;16(12):10321–47. doi: 10.18632/aging.205931 (PMC11236318; doi:10.18632/aging.205931)
Supplement: Supplementary Tables 2 and 3 [file aging-16-205931-s003.pdf]

Supplementary Table 2. 4 risk model genes and their related coefficients.

Id	Coef
NODAL	-0.247060124481458
NDRG2	-0.272265942986441
CCR2	-0.213931600877348
ADAMTS3	0.115299796358193

Supplementary Table 3. PCR primers sequences.

ADAMTS3	Forward	5'ACTGCACCAAAACCTGTGGA 3'
	Reverse	5'CCGACTCAGGCTTTTCACCA 3'
CCR2	Forward	5'AGAGGTCTCGGTTGGGTTGT 3'
	Reverse	5'ATCATAACGTTCTGGGCACC 3'
NDRG2	Forward	5'TACGTCGGCCGTGTCTAT3'
	Reverse	5'GAACTGTGATCCGTGTAGG3'
NODAL	Forward	5'CTGGGAGAACAGGGTACGATAACC5'
	Reverse	5'CGTAGAAGAGGAGGGTCGGG5'
β-Actin gene	Forward	5'TTCCTGGGCATGGAGTCCT3'
	Reverse	5'AGGAGGAGCAATGATCTTGATC3'
